# Supplementary material for: D-2-hydroxyglutarate impairs DNA repair through epigenetic reprogramming
Source: Nat Commun. 2025 Feb 7;16:1431. doi: 10.1038/s41467-025-56781-2 (PMC11806014; doi:10.1038/s41467-025-56781-2)
Supplement: Supplementary file 1 — Supplementary Information [file 41467_2025_56781_MOESM1_ESM.pdf]

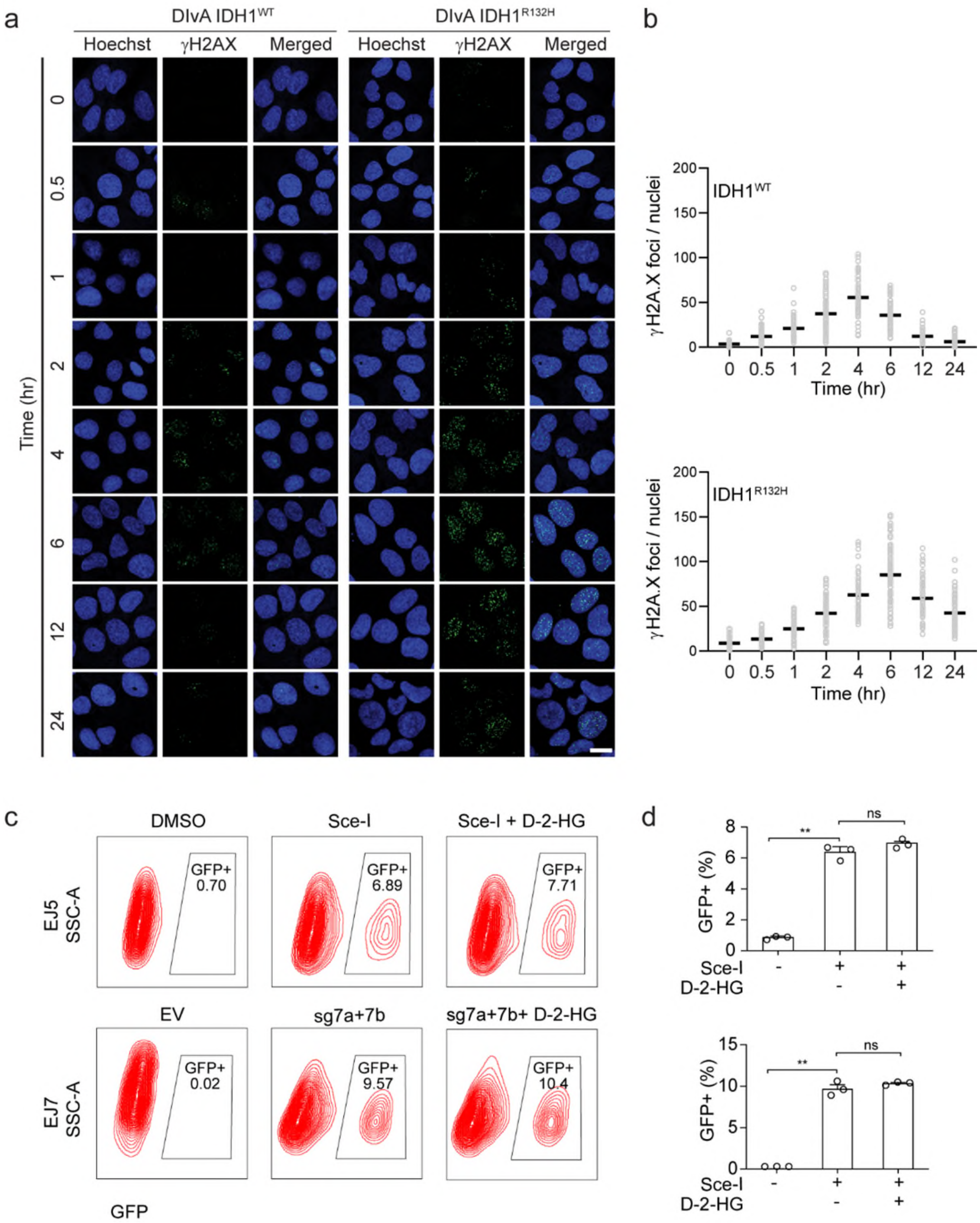

Supplementary Figure 1. DNA damage and NHEJ in IDH1 mutant cells

a. Immunofluorescence staining shows changes of γH2A.X puncta in DIvA cells exposed to 4-OHT (300 nM). Scale bar: 15 μm.

b. Quantification of γH2A.X puncta numbers shown in a.

c. Flowcytometry analysis using EJ5/EJ7 reporter cell lines to measure NHEJ efficiency.

d. Quantification of NHEJ GFP signal shown in c. The statistical significance of differences among groups was tested using the one-way analysis of variance (ANOVA). **$p < 0.01$. EJ5, Sce- vs Sce +, p <0.0001; Sce- vs Sce + D-2-HG, p <0.0001; D-2-HG- vs D-2-HG+, p = 0.1959. EJ-7, Sce- vs Sce +, p <0.0001; Sce- vs Sce + D-2-HG, p <0.0001; D-2-HG- vs D-2-HG+, p = 0.3358.Three biological replicates were performed.

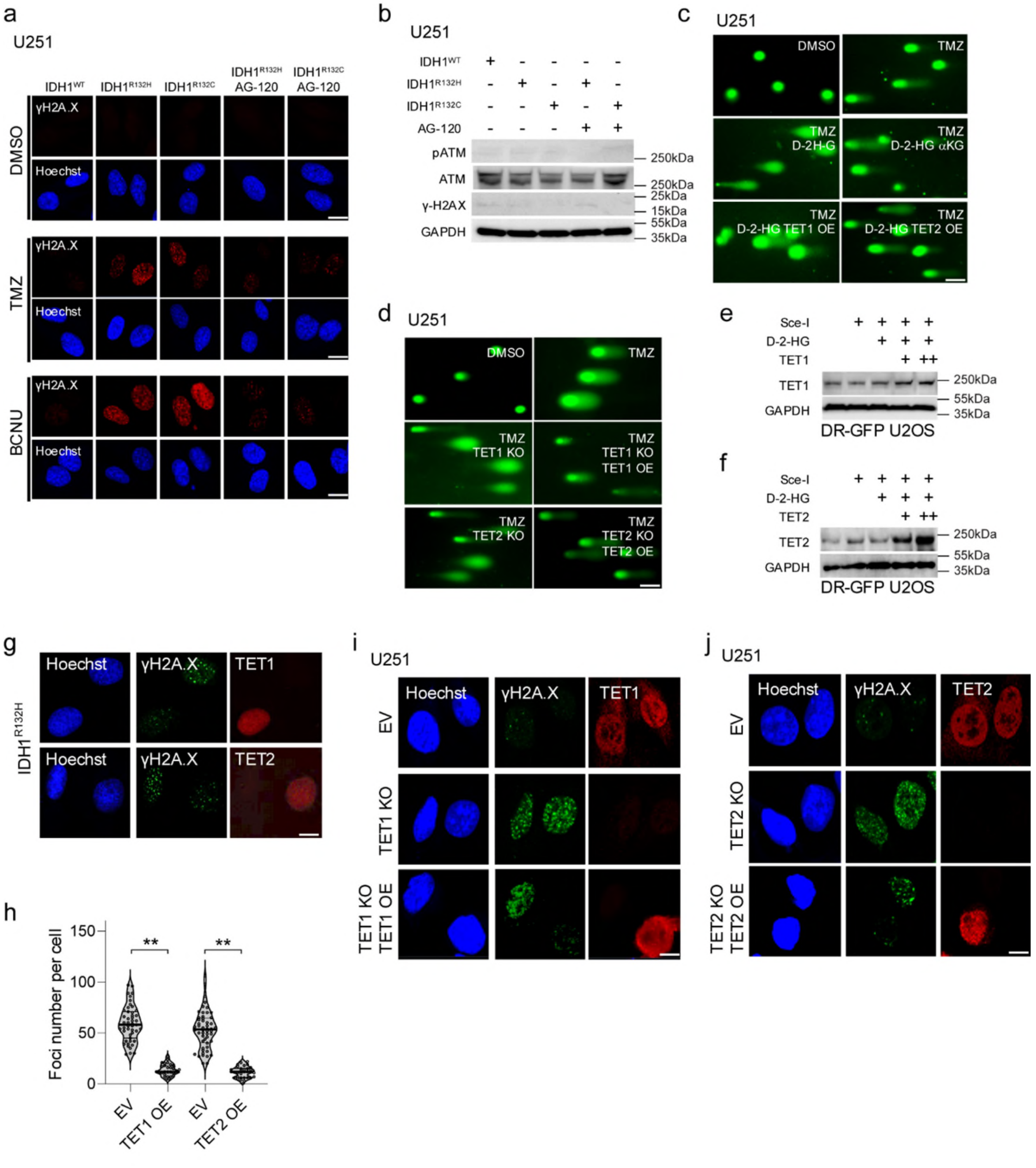

Supplementary Figure 2. TET1 or TET2 overexpression counteracts D-2-HG impact on DDR

a, Immunofluorescence staining shows DDR response in U251 cells. Cells were treated with 150 μM TMZ or 150 μM BCNU for 24 hr. γH2A.X antibodies were used to highlight DNA damage. Scale bar: 15 μm.

b, Western blotting shows ATM phosphorylation and modification of γH2A.X in $IDH1^{WT}$, $IDH1^{R132H}$ and $IDH1^{R132C}$ cells.

c, Representative comet assay images for Fig. 1f. Scale bar: 150 μm.

d, Representative comet assay images for Fig. 1g. Scale bar: 150 μm.

e, Western blotting shows TET1-DDK expression DRGFP U2OS cells.

f, Western blotting assay shows TET2-DDK expression DRGFP U2OS cells.

g, Immunofluorescence staining shows γH2A.X and TET1/2 expression. The $IDH1^{R132H}$ U251 cells were transfected with pCMV6-DDK-TET1 or TET2 plasmids. The cells were treated with 150 μM TMZ for 24 hr. Scale bar: 10 μm.

h, Quantification of γH2A.X puncta in g. The statistical significance of differences among groups was tested using the one-way analysis of variance (ANOVA). $^{**}p < 0.01$. EV vs TET1 OE, $p < 0.0001$; EV vs TET2 OE, $p < 0.0001$; Three biological replicates were performed.

i, Representative γH2A.X and TET1 immunofluorescence staining for Fig. 1m. Scale bar: 10 μm.

j, Representative γH2A.X and TET2 immunofluorescence staining for Fig. 1n. Scale bar: 10 μm.

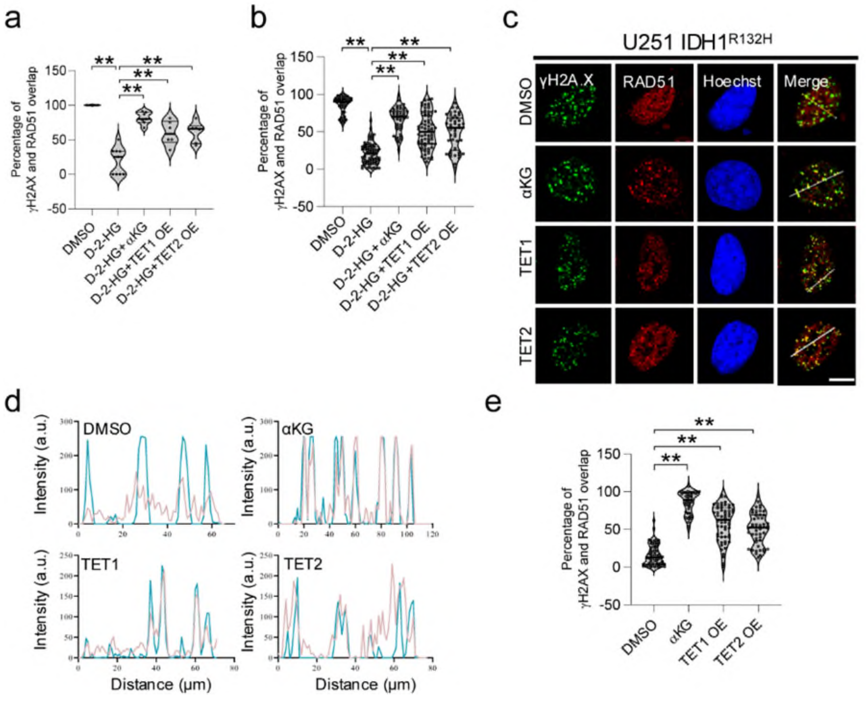

Supplementary Figure 3. TET1 or TET2 overexpression counteracts D-2-HG impact on DDR

a, Immunofluorescence staining in DRGFP U2OS cells shown in Fig. 2e. The statistical significance of differences among groups was tested using the one-way analysis of variance (ANOVA). $**p < 0.01$. DMSO vs. D-2-HG, p <0.0001; D-2-HG vs. D-2-HG+αKG, p <0.0001; D-2-HG vs. D-2-HG+TET1 OE, p =0.0002; D-2-HG vs. D-2-HG+TET2 OE, p =0.0001. Three biological independent experiments were performed for statistical analysis.

b, Immunofluorescence staining for Fig. 2f. The statistical significance of differences among groups was tested using the one-way analysis of variance (ANOVA). $**p < 0.01$. All the indicated p <0.0001. Three biological independent experiments were performed for statistical analysis.

c, Immunofluorescence staining shows TET1 or TET2 overexpression or αKG facilitated RAD51 recruitment to TMZ-induced DNA damage sites in IDH1 mutant U251 cells. The cells were transfected with pCMV6-DDK-TET1 or TET2 plasmids. Then the cells were treated with 150 μM TMZ w/o 10 mM αKG for 24 hr. DNA damage was indicated by γH2A.X. Scale bar: 5 μm.

d, Line profile analysis shows colocalization between γH2A.X and RAD51 puncta in c. Red, RAD51; cyan, γH2A.X.

e, Quantification of the colocalization of RAD51 and γH2A.X puncta in c. The statistical significance of differences among groups was tested using the one-way analysis of variance (ANOVA). $**p < 0.01$. All the indicated p <0.0001. Three biological independent experiments were performed for statistical analysis.

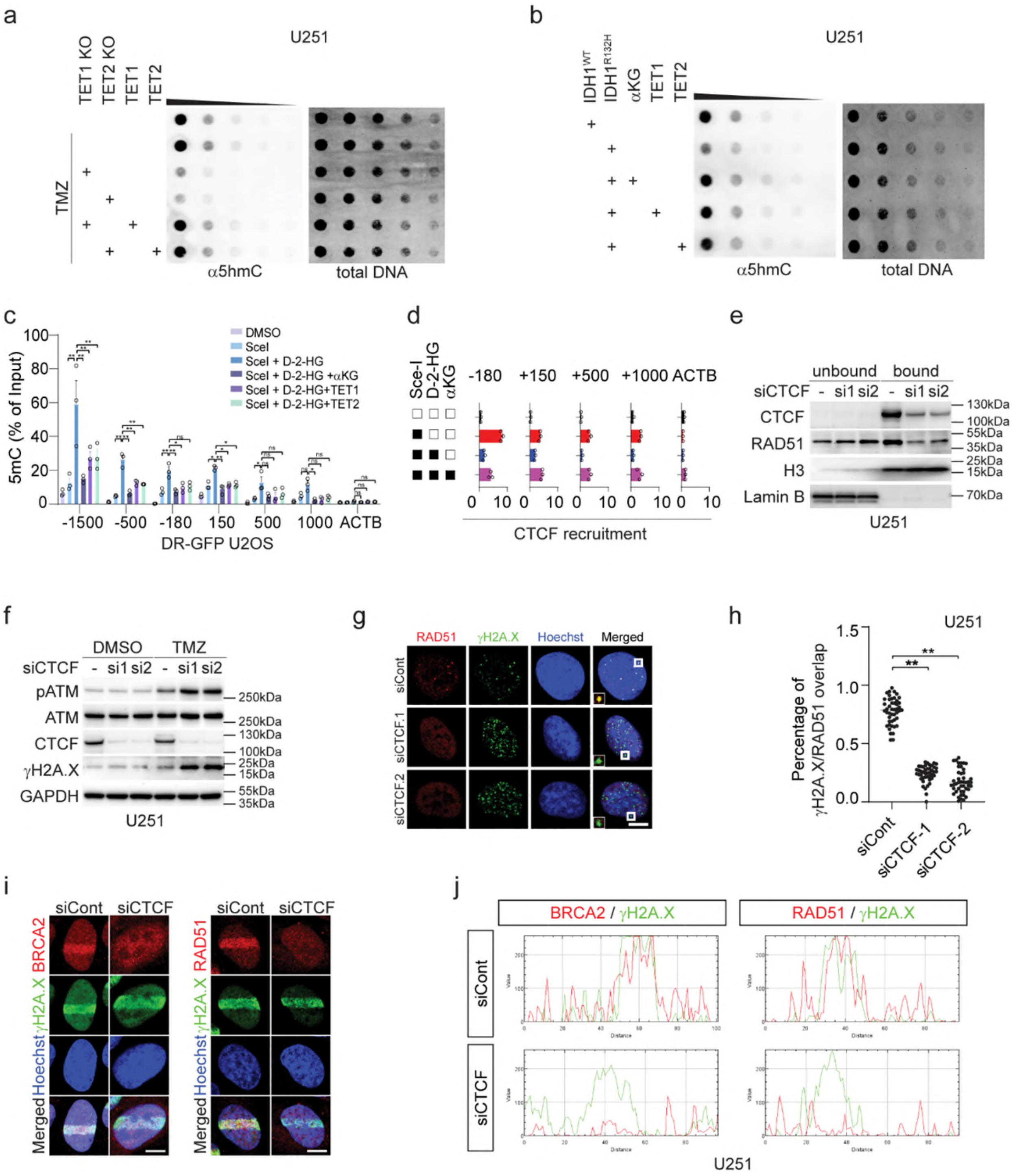

Supplementary Figure 4. 5hmC facilitates CTCF functions in DDR

a, b, Dot blot assay.

c, MeDIP-PCR. **p < 0.01. ns: non-significant. -1500, Sce-I- vs Sce-I+, $p < 0.0001$; Sce-I+ vs D-2-HG, $p < 0.0001$; D-2-HG vs D-2-HG+αKG, $p < 0.0001$; D-2-HG vs D-2-HG+TET1, $p < 0.0001$; D-2-HG vs D-2-HG+TET2, $p < 0.0001$; -500, Sce-I- vs Sce-I+, $p < 0.0001$; Sce-I+ vs D-2-HG, $p < 0.0001$; D-2-HG vs D-2-HG+αKG, $p < 0.0001$; D-2-HG vs D-2-HG+TET1, $p = 0.0017$; D-2-HG vs D-2-HG+TET2, $p = 0.0017$; -180, Sce-I- vs Sce-I+, $p < 0.0001$; Sce-I+ vs D-2-HG, $p = 0.0056$; D-2-HG vs D-2-HG+αKG, $p = 0.0064$; D-2-HG vs D-2-HG+TET1, $p = 0.0352$; D-2-HG vs D-2-HG+TET2, $p = 0.0690$; 150, Sce-I- vs Sce-I+, $p = 0.0001$; Sce-I+ vs D-2-HG, $p = 0.0253$; D-2-HG vs D-2-HG+αKG, $p = 0.0047$; D-2-HG vs D-2-HG+TET1, $p = 0.0319$; D-2-HG vs D-2-HG+TET2, $p = 0.0327$; 500, Sce-I- vs Sce-I+, $p = 0.0071$; Sce-I+ vs D-2-HG, $p = 0.0446$; D-2-HG vs D-2-HG+αKG, $p = 0.1108$; D-2-HG vs D-2-HG+TET1, $p = 0.1158$; D-2-HG vs D-2-HG+TET2, $p = 0.2849$; 1000, Sce-I- vs Sce-I+, $p = 0.0344$; Sce-I+ vs D-2-HG, $p = 0.2424$; D-2-HG vs D-2-HG+αKG, $p = 0.0488$; D-2-HG vs D-2-HG+TET1, $p = 0.0751$; D-2-HG vs D-2-HG+TET2, $p = 0.1499$; ACTB, all the $p > 0.9999$; Group differences were tested with one-way ANOVA. Data are mean ± SEM from three independent experiments.

d, ChIP-PCR. Cells were pretreated with D-2-HG (0.5 mM) and αKG (10 mM) for 24 hr. Sce-I was induced by Shield1 (500 μM) and TA (100 nM).

e, Western blotting.

f, Western blotting. Cells were treated with 150 μM TMZ for 24 hr.

g, Immunofluorescence staining. Cells were treated with 150 μM TMZ for 24 hr. Scale bar: 5 μm.

h, Quantification for the data shown in g. siCR vs. siCTCF-1, $p < 0.0001$; siCR vs. siCTCF, $p < 0.0001$. Group differences were tested with one-way ANOVA. Data are mean ± SEM from three independent experiments.

i, Immunofluorescence. Scale bar: 5 μm.

j, Line profile analysis for i.

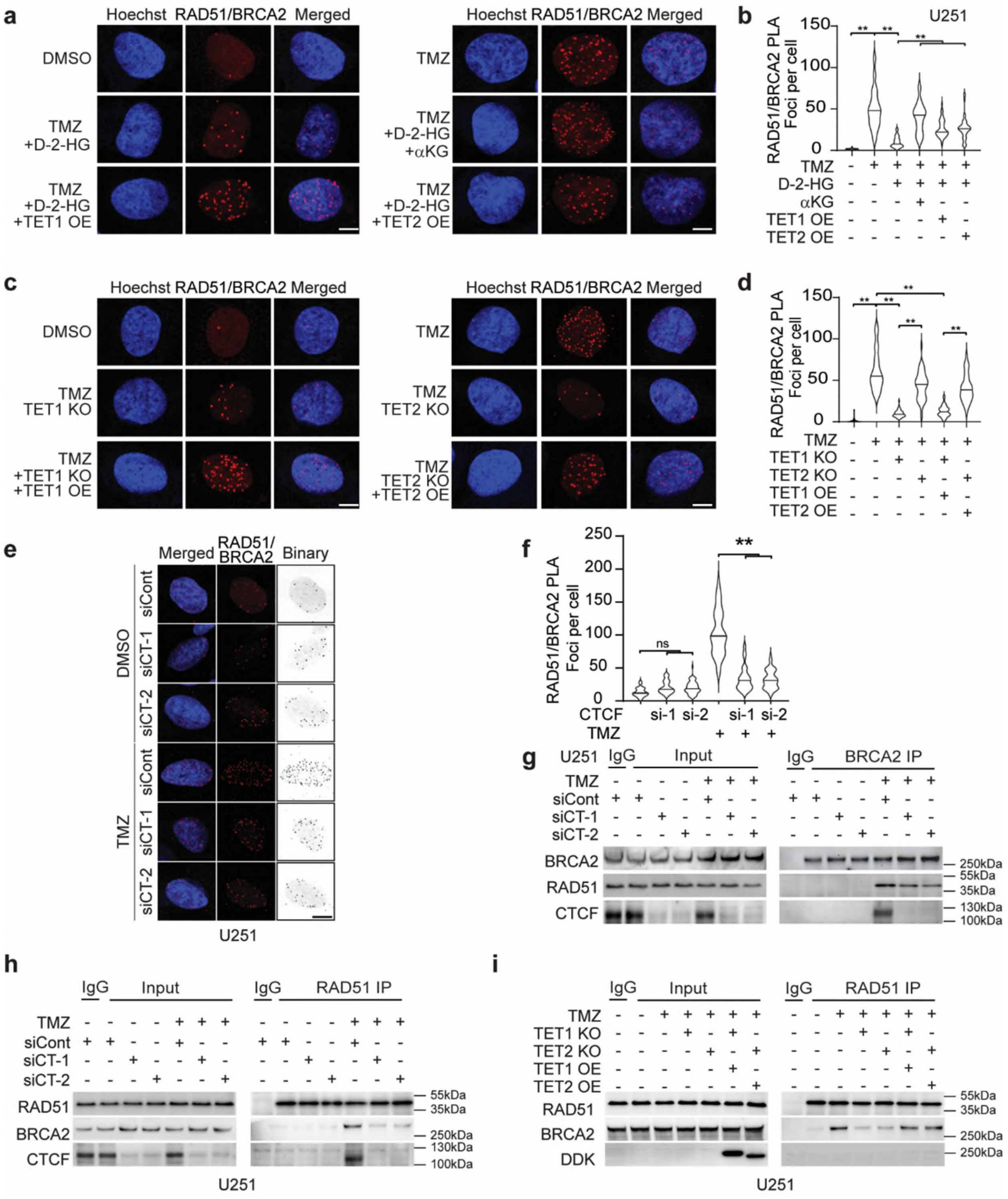
a
Hoechst
RAD51/BRCA2
Merged
DMSO
TMZ
+D-2-HG
+TET1 OE
+αKG
+TET2 OE
b
U251
RAD51/BRCA2 PLA
Foci per cell
150
100
50
0
TMZ
D-2-HG
αKG
TET1 OE
TET2 OE
**
c
TET1 KO
TET2 KO
d
e
RAD51/
BRCA2
Binary
DMSO
siCont
siCT-1
siCT-2
U251
f
250
200
ns
CTCF
si-1
si-2
g
IgG
Input
BRCA2 IP
BRCA2
RAD51
CTCF
250kDa
55kDa
35kDa
130kDa
100kDa
h
RAD51 IP
i
DDK

Supplementary Figure 5. TET1, TET2 and CTCF facilitate HR DNA repair complex assembly

a, PLA assay shows the interactions between BRCA2 and RAD51. The U251 cells were treated with D-2-HG (0.5 mM), αKG (10 mM) or transfected with plasmids expressing TET1 or TET2. Cells were treated with TMZ (150 μM) for 24 hr. Scale bar: 5 μm.

b, Quantification of the PLA foci number shown in a. The statistical significance of differences among groups was tested using the one-way analysis of variance (ANOVA). $^{**}p < 0.01$. All indicated $p < 0.0001$. Three biological replicates were performed.

c, PLA assay shows the interactions between BRCA2 and RAD51 in TET1 or TET2 knock-out U251 cells. Cells were treated with TMZ (150 μM) for 24 hr. Scale bar: 5 μm.

d, Quantification of the PLA foci shown in c. The statistical significance of differences among groups was tested using the one-way analysis of variance (ANOVA). $^{**}p < 0.01$. All indicated $p < 0.0001$. Three biological replicates were performed.

e, PLA assay. Fifty pico mole of CTCF siRNAs were transfected to the cells for 24 hr. Cells were treated with TMZ (150 μM) for 24 hr. Scale bar: 5 μm.

f, Quantification of the PLA foci number shown in e. The statistical significance of differences among groups was tested using the one-way analysis of variance (ANOVA). $^{**}p < 0.01$. All indicated $p < 0.0001$. Three biological replicates were performed.

g, CoIP experiment. Fifty pico mole CTCF siRNAs were transfected to the cells for 24 hr. Cells were then treated with TMZ (150 μM) for 24 hr and proceeded for CoIP using BRCA2 antibody.

h, CoIP. Fifty pico mole CTCF siRNAs were transfected to the cells for 24 hr. Cells were then treated with TMZ (150 μM) for 24 hr and proceeded for CoIP using RAD51 antibody. Immunoprecipitates (IP) and whole-cell extracts (Input) were blotted with the indicated antibodies.

i, CoIP experiment. Cells were transfected with plasmids expressing TET1 or TET2 and were treated TMZ (150 μM) for 24 hr. The lysates were subjected to immunoprecipitation using RAD51 antibody.

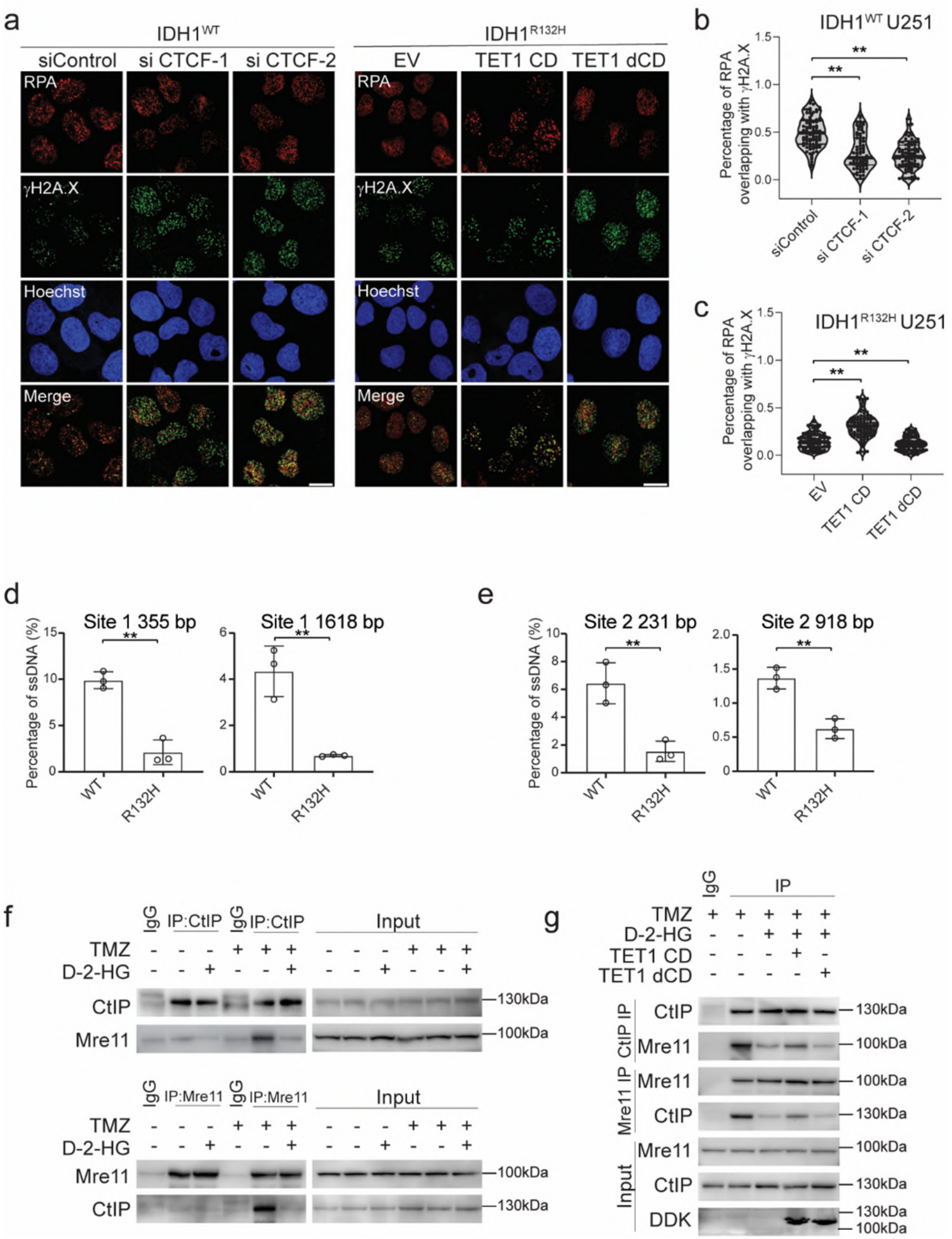

Supplementary Figure 6. End resection in IDH1 mutant cells

a. Immunofluorescence staining of RPA1 and γH2A.X in U251 cells. Cells were treated with TMZ (150 μM) for 24 hr. Scale bar: 15 μm.

b. Quantification of the colocalization of RPA1 and γH2A.X puncta shown IDH1^{WT} U251 cells in a. The statistical significance of differences among groups was tested using the one-way analysis of variance (ANOVA). $^{**}p < 0.01$. All indicated $p < 0.0001$. Three biological replicates were performed.

c. Quantification of the colocalization of RPA1 and γH2A.X puncta shown IDH1^{R132H} U251 cells in a. The statistical significance of differences among groups was tested using the one-way analysis of variance (ANOVA). $^{**}p < 0.01$. All indicated $p < 0.0001$. Three biological replicates were performed.

d. Real-time PCR assay measuring the end resection efficiency in the genomic Site 1 in DIvA cells exposed to 4-OHT (300 nM). The statistical significance of differences between two groups was analyzed using Student t tests. 355 bp, $p = 0.0011$. 1618 bp, $p = 0.0011$. $p = 0.0045$. Three biological replicates were performed.

e. Real-time PCR assay measuring the end resection efficiency in the genomic Site 2 in DIvA cells exposed to 4-OHT (300 nM). The statistical significance of differences between two groups was analyzed using Student t tests. 231 bp, $p = 0.0067$. 918 bp, $p = 0.0011$. $p = 0.0039$. Three biological replicates were performed.

f. CoIP experiment shows interaction between CtIP and Mre11 in U251 cells treated with TMZ (150 μM) and D-2-HG (0.5 mM) for 24 hr. CoIP was conducted using CtIP or Mre11 antibody. Immunoprecipitates (IP) and whole-cell extracts (Input) were blotted with the indicated antibodies.

g. CoIP experiment shows the interaction between CtIP and Mre11 in U251 cells treated with TMZ (150 μM) and TET1 CD/dCD overexpression. CoIP was conducted using CtIP or Mre11 antibody. Immunoprecipitates (IP) and whole-cell extracts (Input) were immunoblotted with the indicated antibodies.

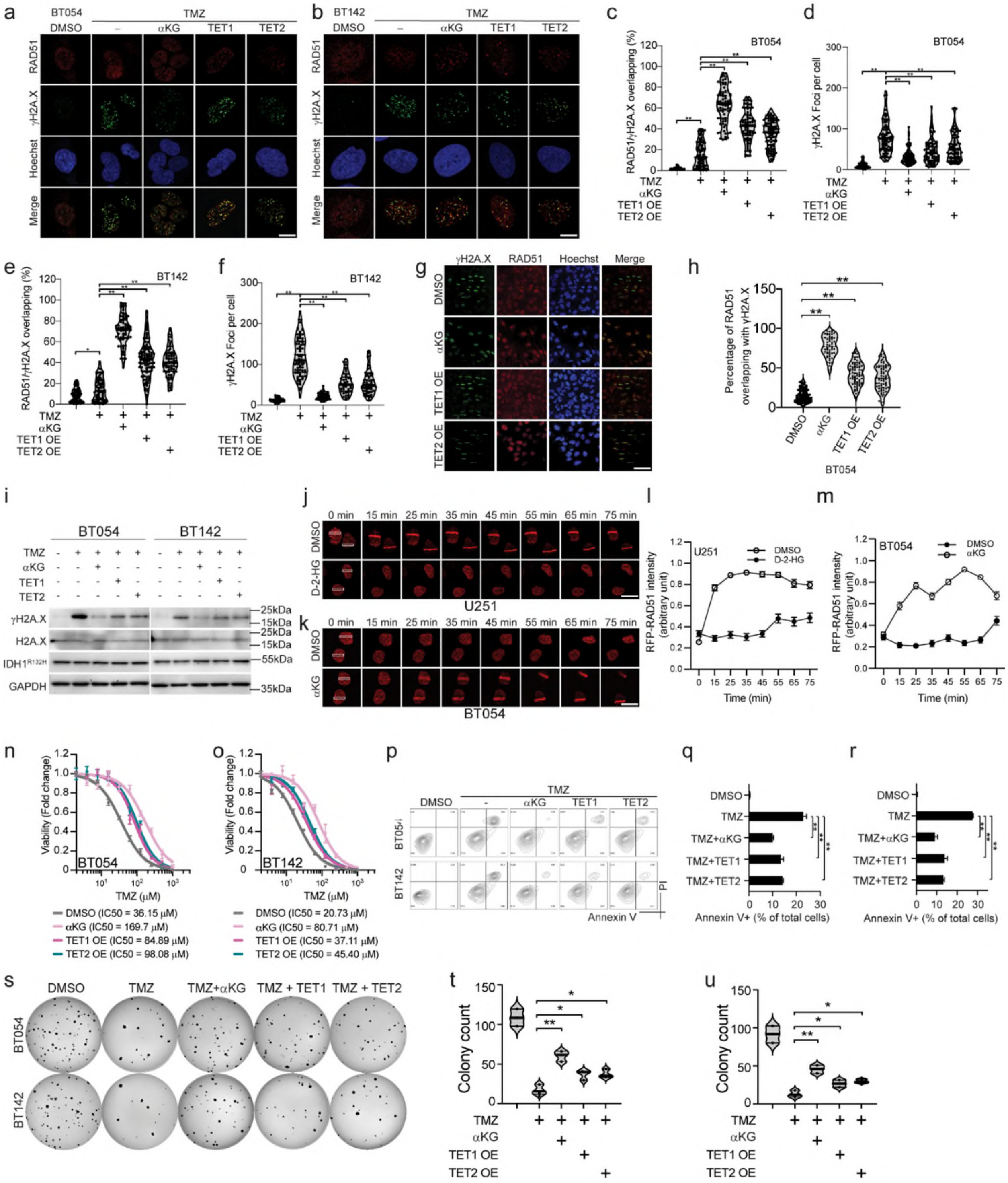

Supplementary Figure 7. DDR deficiency in IDH1 mutant cells

a. Immunofluorescence staining of RAD51 and γH2A.X. Scale bar: 5 μm.

b. Immunofluorescence staining. Scale bar: 5 μm.

c. Quantification for a. All indicated $p < 0.0001$.

d. Quantification for a. All indicated $p < 0.0001$.

e. Quantification of RAD51 and γH2A.X puncta overlapping shown in b. DMSO vs. TMZ, $p = 0.0479$. All other indicated $p < 0.0001$.

f. Quantification for b. All indicated $p < 0.0001$.

g. Laser micro irradiation assay. Scale bar: 50 μm.

h. Quantification for g. All indicated $p < 0.0001$.

i. Western blotting in BT054 and BT142 cells that were treated with TMZ (150 μM), αKG (10 mM), and TET1/2 overexpression.

j. Laser micro irradiation assay shows time course recruitment of RFP-RAD51 in U251 cells treated with D-2-HG (0.5 mM). Scale bar: 20 μm.

k. Laser micro irradiation assay shows time course recruitment of RFP-RAD51 in TB054 cells treated with αKG (10 mM). Scale bar: 20 μm.

l. Quantification of RFP-RAD51 signal shown in j.

m. Quantification of RFP-RAD51 signal shown in k.

n. Dose response. Cells were treated with αKG (10 mM), and TET1/2 overexpression.

o. Dose response.. Cells were treated with αKG (10 mM), and TET1/2 overexpression.

p. Flowcytometry. Cells treated with TMZ (150 μM), αKG (10 mM), and TET1/2 overexpression.

q. Quantification for p. TMZ vs. TMZ+αKG, $p = 0.0003$; TMZ vs. TMZ+TET1 OE, $p = 0.0028$; TMZ vs. TMZ+TET2 OE, $p = 0.0051$. n=3.

r. Quantification for p. All indicated p < 0.0001. n=3.

s. Colony formation assay. n=3.

t. Quantification of BT054 colony number shown in s. TMZ vs. TMZ+αKG, $p = 0.0001$; TMZ vs. TMZ+TET1 OE, $p = 0.0242$; TMZ vs. TMZ+TET2 OE, $p = 0.0206$. Group differences were tested with one-way ANOVA. Data are mean ± SEM. n=3.

u. Quantification for s. TMZ vs. TMZ+αKG, $p = 0.0007$; TMZ vs. TMZ+TET1 OE, $p = 0.0335$; TMZ vs. TMZ+TET2 OE, $p = 0.0129$. Group differences were tested with one-way ANOVA. Data are mean ± SEM. Data are mean ± SEM. n=3.

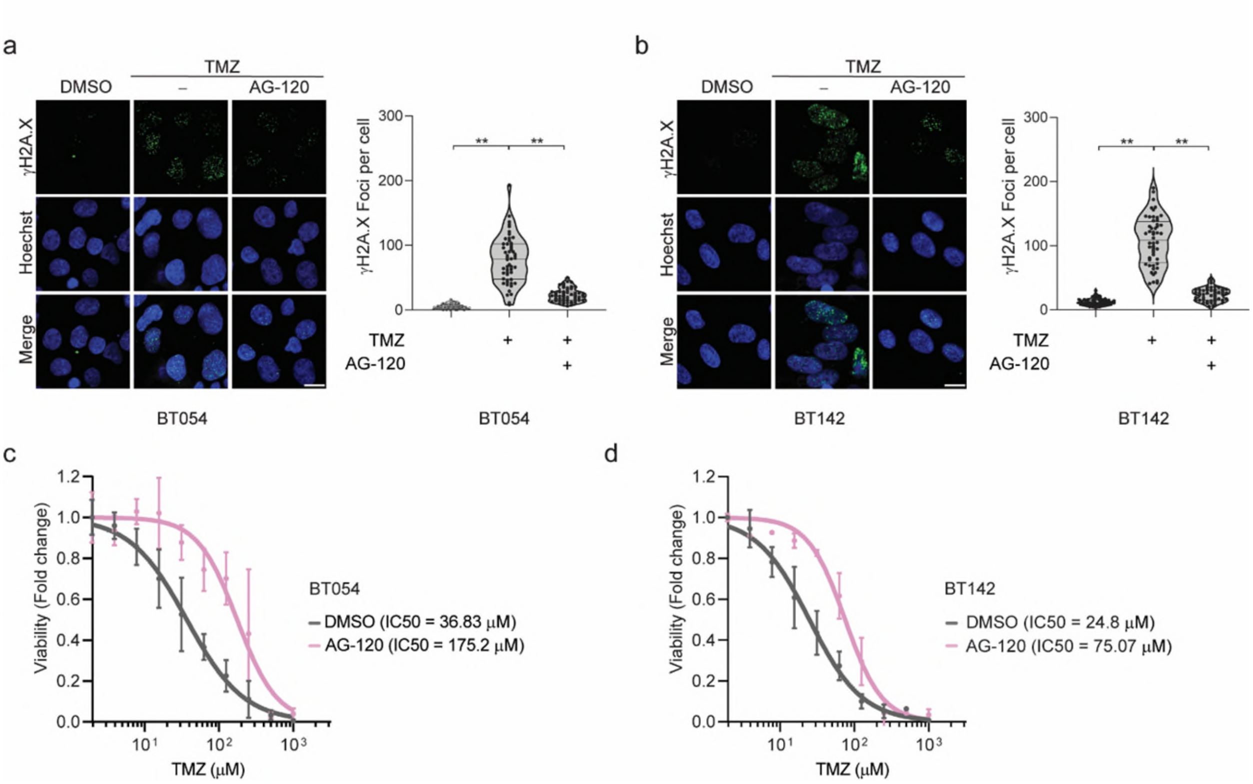

Supplementary Figure 8. AG120 relieves DNA damage in IDH1 mutant cells

a. Immunofluorescence staining shows γH2A.X puncta in BT054 cells treated with TMZ (150 μM), AG-120 (0.5 μM) for 24 hr. The statistical significance of differences among groups was tested using the one-way analysis of variance (ANOVA). $p < 0.0001$. Three biological replicates were performed. Scale bar: 20 μm.

b. Immunofluorescence staining shows RAD51 and γH2A.X puncta in BT142 cells treated with TMZ (150 μM), AG-120 (0.5 μM) for 24 hr. The statistical significance of differences among groups was tested using the one-way analysis of variance (ANOVA). $p < 0.0001$. Three biological replicates were performed. Scale bar: 20 μm.

c. Dose response analysis shows BT054 cellular responses to TMZ. Cells were treated with TMZ with or without AG-120 (0.5 μM).

d. Dose response analysis shows BT142 cellular responses to TMZ. Cells were treated with TMZ with or without AG-120 (0.5 μM).

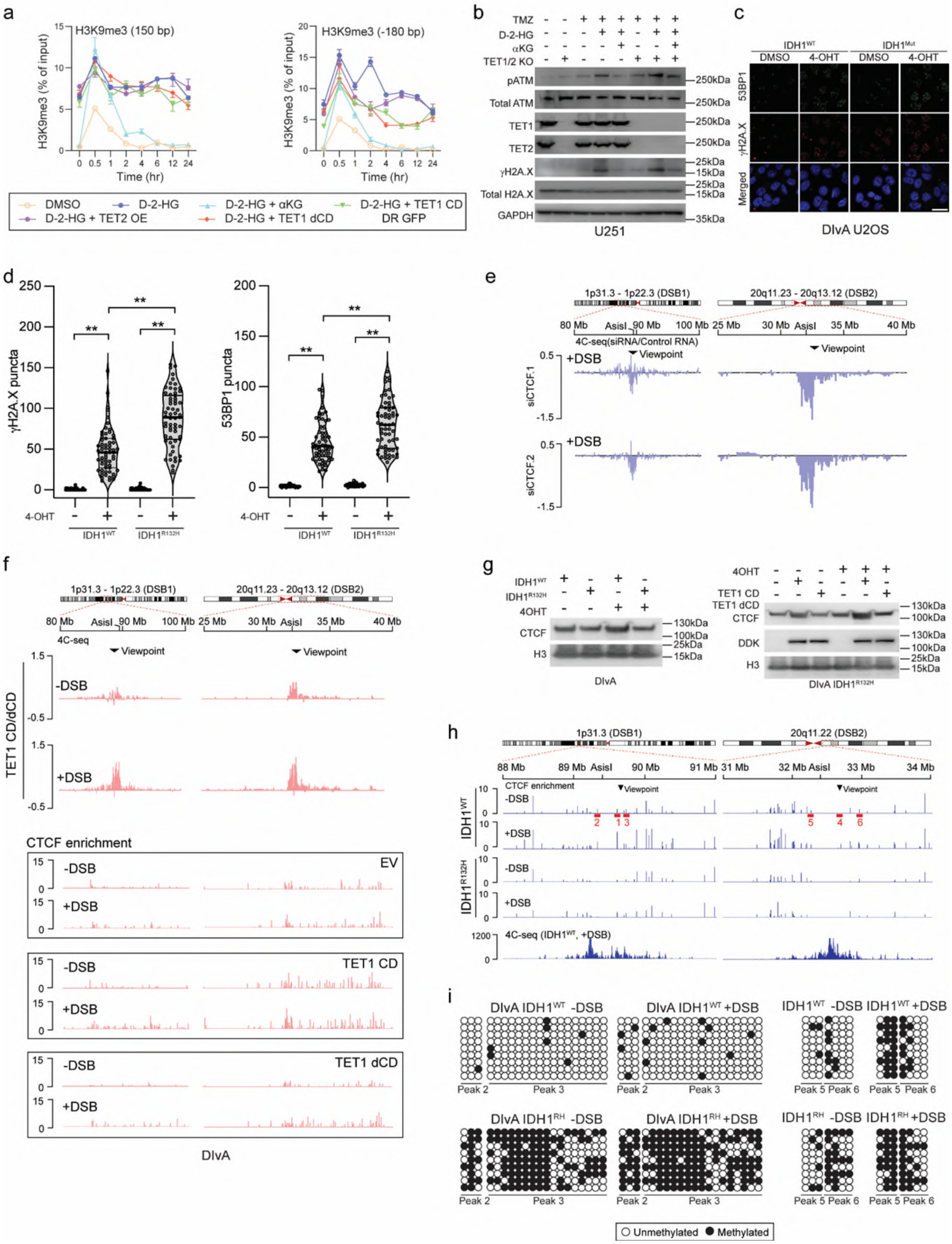
a
H3K9me3 (150 bp)
H3K9me3 (-180 bp)
H3K9me3 (% of input)
Time (hr)
DMSO
D-2-HG
D-2-HG + αKG
D-2-HG + TET1 CD
D-2-HG + TET2 OE
D-2-HG + TET1 dCD
DR GFP
b
TMZ
D-2-HG
αKG
TET1/2 KO
pATM
Total ATM
TET1
TET2
γH2A.X
Total H2A.X
GAPDH
250kDa
25kDa
15kDa
35kDa
U251
c
IDH1WT
IDH1Mut
DMSO
4-OHT
53BP1
γH2A.X
Merged
DIvA U2OS
d
γH2A.X puncta
53BP1 puncta
4-OHT
IDH1WT
IDH1R132H
e
1p31.3 - 1p22.3 (DSB1)
20q11.23 - 20q13.12 (DSB2)
80 Mb
AsisI
90 Mb
100 Mb
25 Mb
30 Mb
35 Mb
40 Mb
4C-seq(siRNA/Control RNA)
Viewpoint
+DSB
siCTCF.1
siCTCF.2
f
4C-seq
TET1 CD/dCD
-DSB
+DSB
CTCF enrichment
EV
TET1 CD
TET1 dCD
DIvA
g
IDH1WT
IDH1R132H
4OHT
CTCF
H3
130kDa
100kDa
DIvA
TET1 CD
TET1 dCD
DDK
DIvA IDH1R132H
h
1p31.3 (DSB1)
20q11.22 (DSB2)
88 Mb
89 Mb
90 Mb
91 Mb
31 Mb
32 Mb
33 Mb
34 Mb
CTCF enrichment
IDH1WT
IDH1R132H
4C-seq (IDH1WT, +DSB)
i
DIvA IDH1WT -DSB
DIvA IDH1WT +DSB
IDH1WT -DSB
IDH1WT +DSB
DIvA IDH1RH -DSB
DIvA IDH1RH +DSB
IDH1RH -DSB
IDH1RH +DSB
Peak 2
Peak 3
Peak 5
Peak 6
Unmethylated
Methylated

Supplementary Figure 9. TET CD facilitates chromatin contacts during DDR

a. ChIP-PCR assay measuring H3K9me3 enrichment at the DSB locus in DRGFP cells. Cells were treated with D-2-HG (0.5 mM), αKG (10 mM), overexpression of TET CD/dCD, or TET2. Three biological replicates were performed.

b. Western blotting shows changes of ATM phosphorylation and γH2A.X levels in U251 TET1/2 KO cells treated with TMZ (150 μM), αKG (10 mM) for 24 hr.

c. Immunofluorescence staining shows γH2A.X and 53BP1 puncta in DIvA cells treated with 4-OHT (300 nM) for 4 hr. Scale bar: 50 μm.

d. Quantification of γH2A.X and 53BP1 puncta shown in c. The statistical significance of differences among groups was tested using the one-way analysis of variance (ANOVA). $p < 0.0001$.

e. Genomic tracks show differential 4C-seq signal (log_2[siCTCF/siControl]) after DSB induction obtained in DIvA cells treated with CTCF RNA interference. Two biological replicates were performed.

f. Top, genomic tracks show differential 4C-seq signal (log2[TET CD/dCD]) in DIvA IDH1^{R132H} cells overexpressing TET1 CD/dCD. Cells were treated with 4-OHT (300 nM) for 4 hr. Bottom, CTCF enrichment at the same genomic loci. Two biological replicates were performed.

g. Western blotting shows changes in chromatin bound CTCF in DIvA cells overexpressing TET1 CD/dCD and treated with 4-OHT (300 nM) for 4 hr.

h. Enlarged genomic tracks show locations of bisulfite PCR amplicons. Peaks 1 and 4 were used in Fig. 5B.

i. Bisulfite PCR assay measures CpG island methylation at the CTCF binding sites. Peaks 2, 3, 5, and 6 were shown.

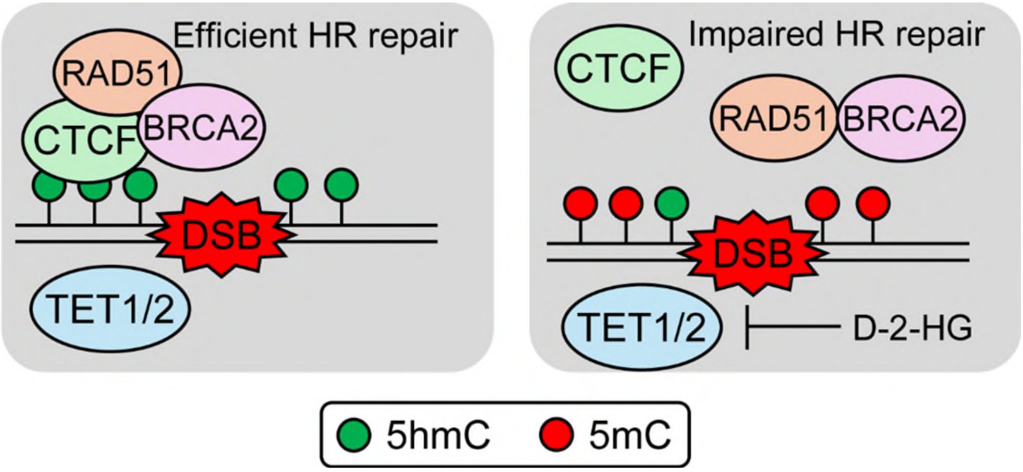

Supplementary Figure 10. D-2-HG establishes "BRCAness" through DNA hypermethylation

Chromatin conformation adjustment is necessary for efficient DNA repair. The presence of IDH mutation and oncometabolite D-2-HG compromise chromatin conformation changes and DNA repair.

Supplementary Table 1: Primers used in this study

Primer name	sequence
Sce-1 1F -180bp	CACCTGCCCCATCTGCTA
Sce-1 1R -180bp	AAGTGCCTCAGCTCCAGGT
Sce-1 1F +150bp	CATGCCCGAAGGCTACGT
Sce-1 1R +150bp	CGGCGCGGGTCTTGTA
AsisI-4C-DSB1.F	TACACGACGCTCTTCCGATCTAACCTGGCAACTTATGAATCAGGA
AsisI-4C-DSB1.R	ACTGGAGTTCAGACGTGTGCTCTTCCGATCTATGTCAAAAGCCAAGGGGACA
AsisI-4C-DSB2.F	TACACGACGCTCTTCCGATCTGGTTATACTAAGATGTCAGTTCCT
AsisI-4C-DSB2.R	ACTGGAGTTCAGACGTGTGCTCTTCCGATCTCACGCACCTGGTTTAGATT
END resection Site2-335-F	GAATCGGATGTATGCGACTGATC
END resection Site2-335-R	TTCCAAAGTTATTCCAACCCGAT
END resection Site2-1618-F	TGAGGAGGTGACATTAGAACTCAGA
END resection Site2-1618-R	AGGACTCACTTACACGGCCTTT
END resection Site2-231-F	ACCATGAACGTGTTCCGAAT
END resection Site2-231-R	GAGCTCCGCAAAGTTTCAAG
END resection Site2-918-F	ACAGATCCAGAGCCACGAAA
END resection Site2-918-R	CCCACTCTCAGCCTTCTCAG
sgRNA TET1.1.F	CACCGGGTCGTAGCCAAATCCAAAA
sgRNA TET1.1.R	AAACTTTTGGATTTGGCTACGACCC
sgRNA TET1.2.F	CACCGCGCCATTGTAAACCCATTGC
sgRNA TET1.2.R	AAACGCAATGGGTTTACAATGGCGC
sgRNA TET2.1.F	CACCGGGATAGAACCAACCATGTTGAGG
sgRNA TET2.1.R	AAACCCTCAACATGGTTGGTTCTATCCC
sgRNA TET2.2.F	CACCGACCGCAATGGAAACACAATCTGG
sgRNA TET2.2.R	AAACCCAGATTGTGTTTCCATTGCGGTC

Supplementary Notes

CTCF binding sites in Figure 5 and Supplementary Fig. 9

The regions highlighted in bold represent the boundaries of the PCR fragment utilized in the bisulfite assay.

CTCF motif is predicted based on “CTCFBSDB 2.0: A database for CTCF binding sites and genome organization”:

DSB1 site 1,

TCATTTACATATTCCGCAGTTTGAACAGAGGAGTGTAAAGCAATTCCAGCAGCCGCAGCAGTACCTGTGACTGTAAT
AAGGCCCATAATGACCGTTATAAGGGTAAAAATAAATCTTTTTGTTTTGGTAAACAGTCCCCTTAACATTTCTGTGA
CAATATGAATGGAAGGAGAGGCTCCCCAAGGTCTATTGAGGGAAACCGGTATCCAAACTCCTTCTCTAGCCCTCACC
AGCAACACAGATGTTTTTACACCAAATGTGCAATCAATACAGATGAAAAGGCAACAGTTTTGACAAGTAATGGTTTG
AGAATTTGGTCGAATATTAATATTTCTGACAATTAACATT**AAAGGAGGCTTGAC**

Motif PWM	Motif Sequence	Input Sequence Name	Motif Start Location	Motif Length	Motif Orientation	Score
EMBL_M1	CTCCTTCTCTAGCC	TCATTTACATATTCCGCAGTTTGAACAGAGGAGTGTAAAGCAATTCCAGCAGCCGCAGCAGTACCTGTGACTGTAATAAGGCCCATAATGACCGTTATAA	111	14	+	-1.51892
EMBL_M2	TGTGTTGCT	TCATTTACATATTCCGCAGTTTGAACAGAGGAGTGTAAAGCAATTCCAGCAGCCGCAGCAGTACCTGTGACTGTAATAAGGCCCATAATGACCGTTATAA	131	9	-	0.948898
REN_20	AAGGTCTATTGAGGGAAACC	TCATTTACATATTCCGCAGTTTGAACAGAGGAGTGTAAAGCAATTCCAGCAGCCGCAGCAGTACCTGTGACTGTAATAAGGCCCATAATGACCGTTATAA	81	20	+	-19.9896
MIT_LM2	AGGTCTATTGAGGGAAACC	TCATTTACATATTCCGCAGTTTGAACAGAGGAGTGTAAAGCAATTCCAGCAGCCGCAGCAGTACCTGTGACTGTAATAAGGCCCATAATGACCGTTATAA	82	19	+	-4.64689
MIT_LM7	ATGGAAGGAGAGGCTCCCCA	TCATTTACATATTCCGCAGTTTGAACAGAGGAGTGTAAAGCAATTCCAGCAGCCGCAGCAGTACCTGTGACTGTAATAAGGCCCATAATGACCGTTATAA	62	20	+	-7.66414
MIT_LM23	TTAACATTAAAGGAGGCTTG	TCATTTACATATTCCGCAGTTTGAACAGAGGAGTGTAAAGCAATTCCAGCAGCCGCAGCAGTACCTGTGACTGTAATAAGGCCCATAATGACCGTTATAA	240	20	+	-8.24391

DSB1 site 2

GCCTCTTGGAAATCCAGCCTTGTCCCAAAACGAGAATATGTAAACATAGC
TGTTTATTAATTCTTTCTCTTTTTCTGTCTTCCTCCCTCTCTCTGTCTCT
GTCTCCCTCTCTGTGTTTCTCTCTGTTAGTTCTGTAGAGAGCGCTTCAAC
TCTTAAAATCCCTCTGCTGCCCTCACGTGGACATCTGCTGAGAAGCACGA
TGTCCTCGAAACTGAAGGCCAAATGGGGGAATTGGTGGAATTATCACCTG
TCTCCCACCAATGGGCTCCTGGTGCTGCAGCCAGGACTTTGACCCACAGT
CACAGCTTTTCCAGG**TCTTTGGCTTAATTTGA**

CTCF motif:

Motif PWM	Motif Sequence	Input Sequence Name	Motif Start Location	Motif Length	Motif Orientation	Score
EMBL_M1	TGCCCTCACGTGGA	GCCTCTTGGAAATCCAGCCTTGTCCCAAAACGAGAATATGTAAACATAGC	117	14	+	9.06496
EMBL_M2	GGTGCTGCA	GCCTCTTGGAAATCCAGCCTTGTCCCAAAACGAGAATATGTAAACATAGC	220	9	+	9.7029
REN_20	ATGTCCACGTGAGGGCAGCA	GCCTCTTGGAAATCCAGCCTTGTCCCAAAACGAGAATATGTAAACATAGC	114	20	-	6.8069
MIT_LM2	TGTCCACGTGAGGGCAGCA	GCCTCTTGGAAATCCAGCCTTGTCCCAAAACGAGAATATGTAAACATAGC	114	19	-	18.3852
MIT_LM7	TTCTCAGCAGATGTCCACGT	GCCTCTTGGAAATCCAGCCTTGTCCCAAAACGAGAATATGTAAACATAGC	124	20	-	8.63347
MIT_LM23	TGGGCTCCTGGTGCTGCAGC	GCCTCTTGGAAATCCAGCCTTGTCCCAAAACGAGAATATGTAAACATAGC	211	20	+	1.46595

DSB1 site 3

ATATAAATGCCAAGTTACTCAGGGACGCACACGTGAGAAGGCTGGGGCCCGACACACGCGGTGTAGTGCCGAAGTCA
CCGGTGTGGCGCGCCTGGGGGACACGCCGAAGCCCATTTCCCTCCCGCTTCTGGAAAGCAGTCCCCGCTCTTTCCAG
CTGCTCCGCGGGGCGTATGGCTGTGCGCTACCTCCTGCGGACCCCAGGCTGGGCGAGGGGGCAGCGATGGCCACATG

GCGCGCTCGGGAGATGGCAGCTGCGCTTTGTTTCCAGCAGTGATAGGTGAGTCACTGGCTCCCGCAGCCCCAGAGC
TGGGCAAACACT

CTCF motif:

Motif PWM	Motif Sequence	Input Sequence Name	Motif Start Location	Motif Length	Motif Orientation	Score
EMBL_M1	CTCCCGCTTCTGGA	ATATAAATGCCAAGTTACTCAGGGACGCACACGTGAGAAGGCTGGGGCCCGACACACGCG	58	14	+	4.95431
EMBL_M2	GGCACTACA	ATATAAATGCCAAGTTACTCAGGGACGCACACGTGAGAAGGCTGGGGCCCGACACACGCG	1	9	-	10.523
REN_20	CTTTCCAGAAGCGGGAGGGA	ATATAAATGCCAAGTTACTCAGGGACGCACACGTGAGAAGGCTGGGGCCCGACACACGCG	55	20	-	-0.553439
MIT_LM2	TTTCCAGAAGCGGGAGGGA	ATATAAATGCCAAGTTACTCAGGGACGCACACGTGAGAAGGCTGGGGCCCGACACACGCG	55	19	-	-0.676437
MIT_LM7	TTTCCAGAAGCGGGAGGGAA	ATATAAATGCCAAGTTACTCAGGGACGCACACGTGAGAAGGCTGGGGCCCGACACACGCG	54	20	-	2.71306
MIT_LM23	TTTCCAGAAGCGGGAGGGAA	ATATAAATGCCAAGTTACTCAGGGACGCACACGTGAGAAGGCTGGGGCCCGACACACGCG	54	20	-	1.60769

DSB4 site 4

AGTTGAGGAGGAGGACAGGGATGCAGAGGGCTGGGGGAGAGACACAAAGAAGGGAGGGAACATGAAAAAAGGGCTGG
GTGGGGGAGTGGGGAAGAAGTGGCTGTGGGCCTAGGTGTCCAGCCCTTTCCTCTTGCTCTGGGTTTTGGAAAAGCCA
CTGACCCTGATTTAGCTGTCTGGT**GGGGCCAAAGAGTCCC**TTCAGTCCCAGGTTCCCTGGGCTGAGACTGCCCAGTT
ACTGGTTTTCCATGTGCTTCCTGGAGCCTGGGTCCTCCCGCACCTCTCCACTAAGGGGCAGCATCACCCAAG**CG**TTC
CGCTGTCCTCGCCGCAGCCCGCAGCCGGGCTGGGAAAGTTGGCCAAGGGTGAGGGGCTGCTCTGCGCTGCGCTTGGC
CCCGCCTTCCCACAGCAACTGCAGGGGTTTGCTCCTTTAGAAATGCACGAATGTCCTTCTCCATCTCTGTGATGGCT
GGAGGGTAGAGGTGGGCTAGGGCGGGGCTGGGGAGTGGAGTTGTGGGGGGATGCAGAAGGCTTGGGGTGTCCCAGGC
TGTGGCTGGGCTCTTGGAGGACCAGCTCAGGCACAGCTTCCAGCTCAGCCCACTGGCAGAGTCC

No motif found

DSB4 site 5

CATGGCTCACTGTAGTCTTGACCTTCTGGGCTCAAGTGATCCTCTCCTTTGGCCTCCTGAATAGCTGGGACCACAGA
CATATGCCACCACATCCAGTTAATATTTTAAAAATATTTTTGTGGAGATGAGGTCTCGCCATGTTGCTCAGGCAAGC
AATCCTCCCGCCTTGGCCTCTCGAAGTGTTGGGATTACAGGTGTGAGCCACTGTGCCCAGCCTGCACTTCTAAATAG
GGGGGGTCAGGAAGGGCTCACTGAGAAGCATGTGAACAAAGACCTGAAGGTGGGGAGGGGTGAGCCGTGCCAATACT
GGGAGAAGAGTATTCCGGGCAGAGGGAGTAGCCAGTGCAAAGGCCCTGAGGCAGAGGTGTGGTTGGAGAGTTTGAGG
AACAGGTAGGAGGCCAGTATGGATGGAGTGGAGTGAGCAAGGAGGTAAGGAATGAAGTTAGAGAGGAAGAGGGGGAC
AGGAGTTGGAGGGACTCATCGGCCATGGCAAGGGGTTTATCTTTT**ACTTGGAGCAAAACTGGAGCCATGG**GAGTGTT
TGGAGCAGAGCGGTCCTTGGTTCAGGTCTGGCTGACCTATCCGTTGAGTGAGGGGACTCCTGGACAATACAGTGTCA
TGGCGCCTCCCGTGGCCATTTTGGGCACTGCACCTTCTCTAGCCTGTGACCCTCCTGGACTGAAGGGGACTTACAGG
CTGCTCAGACACCCTGCAGCTGCCCCC**CTGCCCTGGTGTTG**ACTGGGACTCCCTCTCTTCTGCCCTCTAGAAAAACT
TCATTGCTGTCAGCGCTGCCAACCGCTTCAAGAAGATCAGCAGCTCGGGGGCACTGATGGCTCTGGGGGTCTGAGCC
CTGGGCGCAGCTGAAGCCTGGACGCAGCCACACAGTGGCCGGGGCTGAAGCCACACAGCCCAGAAGGCCAGAAAAGG
CAGCCAGATCCCCAGGGCAGCCTCGTTAGGACAAGGCTGTGCCAGGCTGGGAGGCTCGGGGCTCCCCACGCCCCCAT
GCAGTGACCGCTTCCCCGATGTGAGCCGCCTCGGAGTGTGGCCTGGATCCATCCTGCTAGCACCTCCCCAGACAGGG
CTCCAGCCTGTCGGCCACACCCCAGACTCCAGGCCCCGTTGAAGCCGCTCCCGGTTCCCTCCCCAGCTCCTCGTCT
TTGAACTGCCG

CTCF motif:

Motif PWM	Motif Sequence	Input Sequence Name	Motif Start Location	Motif Length	Motif Orientation	Score
EMBL_M1	CCCCACCTTCAGGT	CATGGCTCACTGTAGTCTTGACCTTCTGGGCTCAAGTGATCCTCTCCTTT	222	14	-	5.8528
EMBL_M2	GGCACTGCA	CATGGCTCACTGTAGTCTTGACCTTCTGGGCTCAAGTGATCCTCTCCTTT	589	9	+	12.8887
REN_20	CCGGCCACTGTGTGGCTGCG	CATGGCTCACTGTAGTCTTGACCTTCTGGGCTCAAGTGATCCTCTCCTTT	819	20	-	-2.29948
MIT_LM2	GTTTTTCTAGAGGGCAGAA	CATGGCTCACTGTAGTCTTGACCTTCTGGGCTCAAGTGATCCTCTCCTTT	700	19	-	3.17696
MIT_LM7	AGACCTGAAGGTGGGGAGGG	CATGGCTCACTGTAGTCTTGACCTTCTGGGCTCAAGTGATCCTCTCCTTT	220	20	+	1.21084
MIT_LM23	AGACCTGAAGGTGGGGAGGG	CATGGCTCACTGTAGTCTTGACCTTCTGGGCTCAAGTGATCCTCTCCTTT	220	20	+	1.24475

DSB4 site 6

GGAGGAAAGTATTCAGTGTTTTCAGAACCACCCACCAGGAGGCGCTTTTGCATCAGGGGCCCAATCAGGTTAGCCCA
GAGCACCTGGATTTAAGTAGCAAAAACAAATGACAATCTCTAGCCCAATGTCTCAAGAACATTCCAGTCTTTTACTT
CATCTCACCCTCGAAACTTCCTTTTTTTTTTTTTAGACAGAGTCTCACTCTGTCACCACGGCTGGAGTGCAGTGGTG
CAATCACAGCTCACTGCAGCGTCGACCTCCCAGACTCAAGCAATCCTCCCACCTCAGCTTCCTAGGTAGCTGGGACT
AGGTGCACA**TCACCATGCCTAGC**

CTCF motif:

Motif PWM	Motif Sequence	Input Sequence Name	Motif Start Location	Motif Length	Motif Orientation	Score
EMBL_M1	TCACCACGGCTGGA	GGAGGAAAGTATTCAGTGTTTTCAGAACCACCCACCAGGAGGCGCTTTTGCATCAGGGGCCCAATCAGGTTAGC	132	14	+	-5.36647
EMBL_M2	AGTGGTGCA	GGAGGAAAGTATTCAGTGTTTTCAGAACCACCCACCAGGAGGCGCTTTTGCATCAGGGGCCCAATCAGGTTAGC	150	9	+	7.57639
REN_20	ACCACGGCTGGAGTGCAGTG	GGAGGAAAGTATTCAGTGTTTTCAGAACCACCCACCAGGAGGCGCTTTTGCATCAGGGGCCCAATCAGGTTAGC	134	20	+	-10.6053
MIT_LM2	CCACGGCTGGAGTGCAGTG	GGAGGAAAGTATTCAGTGTTTTCAGAACCACCCACCAGGAGGCGCTTTTGCATCAGGGGCCCAATCAGGTTAGC	135	19	+	-11.6621
MIT_LM7	TAGGAAGCTGAGGTGGGAGG	GGAGGAAAGTATTCAGTGTTTTCAGAACCACCCACCAGGAGGCGCTTTTGCATCAGGGGCCCAATCAGGTTAGC	201	20	-	-2.80927
MIT_LM23	CTGGGACTAGGTGCACATCA	GGAGGAAAGTATTCAGTGTTTTCAGAACCACCCACCAGGAGGCGCTTTTGCATCAGGGGCCCAATCAGGTTAGC	226	20	+	-10.5571
