# Supplementary material for: D-2-hydroxyglutarate impairs DNA repair through epigenetic reprogramming
Source: Nat Commun. 2025 Feb 7;16:1431. doi: 10.1038/s41467-025-56781-2 (PMC11806014; doi:10.1038/s41467-025-56781-2)
Supplement: Supplementary file 2 — Reporting Summary [file 41467_2025_56781_MOESM2_ESM.pdf]

nature portfolio

Corresponding author(s): Chunzhang Yang

Last updated by author(s): Jan 21, 2025

Reporting Summary

Nature Portfolio wishes to improve the reproducibility of the work that we publish. This form provides structure for consistency and transparency in reporting. For further information on Nature Portfolio policies, see our Editorial Policies and the Editorial Policy Checklist.

Statistics

For all statistical analyses, confirm that the following items are present in the figure legend, table legend, main text, or Methods section.

n/a	Confirmed	
☐	☒	The exact sample size (*n*) for each experimental group/condition, given as a discrete number and unit of measurement
☐	☒	A statement on whether measurements were taken from distinct samples or whether the same sample was measured repeatedly
☐	☒	The statistical test(s) used AND whether they are one- or two-sided<br>*Only common tests should be described solely by name; describe more complex techniques in the Methods section.*
☒	☐	A description of all covariates tested
☒	☐	A description of any assumptions or corrections, such as tests of normality and adjustment for multiple comparisons
☐	☒	A full description of the statistical parameters including central tendency (e.g. means) or other basic estimates (e.g. regression coefficient) AND variation (e.g. standard deviation) or associated estimates of uncertainty (e.g. confidence intervals)
☐	☒	For null hypothesis testing, the test statistic (e.g. *F*, *t*, *r*) with confidence intervals, effect sizes, degrees of freedom and *P* value noted<br>*Give P values as exact values whenever suitable.*
☒	☐	For Bayesian analysis, information on the choice of priors and Markov chain Monte Carlo settings
☒	☐	For hierarchical and complex designs, identification of the appropriate level for tests and full reporting of outcomes
☒	☐	Estimates of effect sizes (e.g. Cohen's *d*, Pearson's *r*), indicating how they were calculated

Our web collection on statistics for biologists contains articles on many of the points above.

Software and code

Policy information about availability of computer code

Data collection	Western blots were scanned using the ChemiDocTM touch imaging system v2.1.0.35.<br>ChIP-qPCR data were collected using the QuantStudio Software v1.3.<br>Immunofluorescence images were captured with ZEN 2011.
Data analysis	- ChIP-qPCR data were analyzed using Microsoft Excel (v16.75.2) and GraphPad Prism 8.4.3.<br>- ChIP-seq data were mapped to the GRCh38 using Bowtie2-2.5.1. Peaks were identified with MACS2 (v2.2.7.1).<br>- For 4C-seq, mapping was done using Pipe4C (R package, R/4.2 version) and peaks were called with PeakC (R package, R/4.2 version).<br>- Flow cytometry data was analyzed with Flowjo v10.7.2.<br>- Statistical Analysis was done in GraphPad Prism 8.4.3.<br>- Figures were compiled using Adobe Illustrator 2024.

For manuscripts utilizing custom algorithms or software that are central to the research but not yet described in published literature, software must be made available to editors and reviewers. We strongly encourage code deposition in a community repository (e.g. GitHub). See the Nature Portfolio guidelines for submitting code & software for further information.

Data

Policy information about availability of data

All manuscripts must include a data availability statement. This statement should provide the following information, where applicable:

- Accession codes, unique identifiers, or web links for publicly available datasets
- A description of any restrictions on data availability
- For clinical datasets or third party data, please ensure that the statement adheres to our policy

Our sequencing data from ChIP-seq and 4C-seq have been deposited in the Gene Expression Omnibus and the accession number is GSE283196 and the reviewer token is: mvglmkimrrczviz

GSE283196 CTCF distribution around AsisI sites in IDHwt and IDHR132H DIvA cells Nov 27, 2025 approved None
GSM8657501 DIvA cell, IDH wt, non treated, CTCF, ChIP, Rep 1 Nov 27, 2025 approved BW NARROWPEAK
GSM8657502 DIvA cell, IDH wt, non treated, CTCF, ChIP, Rep 2 Nov 27, 2025 approved BW NARROWPEAK
GSM8657503 DIvA cell, IDH wt, non treated, CTCF, ChIP, Rep 3 Nov 27, 2025 approved BW NARROWPEAK
GSM8657504 DIvA cell, IDH wt, non treated, CTCF, ChIP, Rep 4 Nov 27, 2025 approved BW NARROWPEAK
GSM8657506 DIvA cell, IDH wt, 4-OHT, CTCF, Rep 1 Nov 27, 2025 approved BW NARROWPEAK
GSM8657507 DIvA cell, IDH wt, 4-OHT, CTCF, Rep 2 Nov 27, 2025 approved BW NARROWPEAK
GSM8657508 DIvA cell, IDH wt, 4-OHT, CTCF, Rep 3 Nov 27, 2025 approved BW NARROWPEAK
GSM8657509 DIvA cell, IDH wt, 4-OHT, CTCF, Rep 4 Nov 27, 2025 approved BW NARROWPEAK
GSM8657510 DIvA cell, IDH mutant, non treated, CTCF, Rep 1 Nov 27, 2025 approved BW NARROWPEAK
GSM8657511 DIvA cell, IDH mutant, non treated, CTCF, Rep 2 Nov 27, 2025 approved BW NARROWPEAK
GSM8657512 DIvA cell, IDH mutant, non treated, CTCF, Rep 3 Nov 27, 2025 approved BW NARROWPEAK
GSM8657513 DIvA cell, IDH mutant, non treated, CTCF, Rep 4 Nov 27, 2025 approved BW NARROWPEAK
GSM8657514 DIvA cell, IDH mutant, 4-OHT,CTCF, Rep 1 Nov 27, 2025 approved BW NARROWPEAK
GSM8657515 DIvA cell, IDH mutant, 4-OHT,CTCF, Rep 2 Nov 27, 2025 approved BW NARROWPEAK
GSM8657516 DIvA cell, IDH mutant, 4-OHT,CTCF, Rep 3 Nov 27, 2025 approved BW NARROWPEAK
GSM8657517 DIvA cell, IDH mutant, 4-OHT,CTCF, Rep 4 Nov 27, 2025 approved BW NARROWPEAK
GSM8657518 DIvA cell, IDH mutant, non treated, TETCD, Rep1 Nov 27, 2025 approved BW NARROWPEAK
GSM8657519 DIvA cell, IDH mutant, non treated, TETCD, Rep2 Nov 27, 2025 approved BW NARROWPEAK
GSM8657520 DIvA cell, IDH mutant, TETCD4-OHT over expression, Rep1 Nov 27, 2025 approved BW NARROWPEAK
GSM8657521 DIvA cell, IDH mutant, TETCD4-OHT over expression, Rep2 Nov 27, 2025 approved BW NARROWPEAK
GSM8657522 DIvA cell, IDH mutant, non treated, TETdCD, Rep1 Nov 27, 2025 approved BW NARROWPEAK
GSM8657523 DIvA cell, IDH mutant, non treated, TETdCD, Rep2 Nov 27, 2025 approved BW NARROWPEAK
GSM8657524 DIvA cell, IDH mutant, non treated, TETdCD, Rep3 Nov 27, 2025 approved BW NARROWPEAK
GSM8657525 DIvA cell, IDH mutant, TETdCD4-OHT over expression, Rep1 Nov 27, 2025 approved BW NARROWPEAK
GSM8657526 DIvA cell, IDH mutant, TETdCD4-OHT over expression, Rep2 Nov 27, 2025 approved BW NARROWPEAK
GSM8657527 DIvA cell, IDH wt, non treated, input, Rep 1 Nov 27, 2025 approved None
GSM8657528 DIvA cell, IDH wt, non treated, input, Rep 2 Nov 27, 2025 approved None
GSM8657529 DIvA cell, IDH wt, non treated, input, Rep 3 Nov 27, 2025 approved None
GSM8657530 DIvA cell, IDH wt, non treated, input, Rep 4 Nov 27, 2025 approved None
GSM8657531 DIvA cell, IDH wt, 4-OHT, input, Rep 1 Nov 27, 2025 approved None
GSM8657532 DIvA cell, IDH wt, 4-OHT, input, Rep 2 Nov 27, 2025 approved None
GSM8657533 DIvA cell, IDH wt, 4-OHT, input, Rep 3 Nov 27, 2025 approved None
GSM8657534 DIvA cell, IDH wt, 4-OHT, input, Rep 4 Nov 27, 2025 approved None
GSM8657535 DIvA cell, IDH mutant, non treated, input, Rep 1 Nov 27, 2025 approved None
GSM8657536 DIvA cell, IDH mutant, non treated, input, Rep 2 Nov 27, 2025 approved None
GSM8657537 DIvA cell, IDH mutant, 4-OHT, input, Rep 1 Nov 27, 2025 approved None
GSM8657538 DIvA cell, IDH mutant, 4-OHT, input, Rep 2 Nov 27, 2025 approved None
GSM8657539 DIvA cell, IDH mutant, non treated, TETCD, input, Rep1 Nov 27, 2025 approved None
GSM8657540 DIvA cell, IDH mutant, non treated, TETCD, input, Rep2 Nov 27, 2025 approved None
GSM8657541 DIvA cell, IDH mutant, TETCD4-OHT over expression, input, Rep1 Nov 27, 2025 approved None
GSM8657542 DIvA cell, IDH mutant, TETCD4-OHT over expression, input, Rep2 Nov 27, 2025 approved None
GSM8657543 DIvA cell, IDH mutant, non treated, TETdCD, input, Rep1 Nov 27, 2025 approved None
GSM8657544 DIvA cell, IDH mutant, non treated, TETdCD, input, Rep2 Nov 27, 2025 approved None
GSM8657545 DIvA cell, IDH mutant, TETdCD4-OHT over expression, input, Rep1 Nov 27, 2025 approved None
GSM8657546 DIvA cell, IDH mutant, TETdCD4-OHT over expression, input, Rep2 Nov 27, 2025 approved None
GSM8657547 DIvA cell, IDH mutant, TETCD4-OHT, over expression, 4C-seq, DSB1, Rep 1 Nov 27, 2025 approved BW
GSM8657548 DIvA cell, IDH mutant, TETCD4-OHT, over expression, 4C-seq, DSB1, Rep 2 Nov 27, 2025 approved BW
GSM8657549 DIvA cell, IDH mutant, 4-OHT, 4C-seq, DSB1, Rep 1 Nov 27, 2025 approved BW
GSM8657550 DIvA cell, IDH mutant, 4-OHT, 4C-seq, DSB1, Rep 2 Nov 27, 2025 approved BW
GSM8657551 DIvA cell, IDH mutant, TETdCD4-OHT, over expression, 4C-seq, DSB1, Rep 1 Nov 27, 2025 approved BW
GSM8657552 DIvA cell, IDH mutant, TETdCD4-OHT, over expression, 4C-seq, DSB1, Rep 2 Nov 27, 2025 approved BW
GSM8657553 DIvA cell, IDH mutant, TETdCD4-OHT, empty vector, 4C-seq, DSB1, Rep 1 Nov 27, 2025 approved BW
GSM8657554 DIvA cell, IDH mutant, TETdCD4-OHT, empty vector, 4C-seq, DSB1, Rep 2 Nov 27, 2025 approved BW
GSM8657555 DIvA cell, IDH mutant, TETdCD4-OHT, Knock down (si1), 4C-seq, DSB1, Rep 1 Nov 27, 2025 approved BW
GSM8657556 DIvA cell, IDH mutant, TETdCD4-OHT, Knock down (si1), 4C-seq, DSB1, Rep 2 Nov 27, 2025 approved BW

GSM8657557	DIvA cell, IDH mutant, TETdCD4-OHT, Knock down (si2), 4C-seq, DSB1, Rep 1	Nov 27, 2025	approved	BW
GSM8657558	DIvA cell, IDH mutant, TETdCD4-OHT, Knock down (si2), 4C-seq, DSB1, Rep 2	Nov 27, 2025	approved	BW
GSM8657559	DIvA cell, IDH mutant, non treated, 4C-seq, DSB1, Rep 1	Nov 27, 2025	approved	None
GSM8657560	DIvA cell, IDH mutant, non treated, 4C-seq, DSB1, Rep 2	Nov 27, 2025	approved	None
GSM8657561	DIvA cell, IDH mutant, non treated, TETCD, over expression, 4C-seq, DSB1, Rep 1	Nov 27, 2025	approved	None
GSM8657562	DIvA cell, IDH mutant, non treated, TETCD, over expression, 4C-seq, DSB1, Rep 2	Nov 27, 2025	approved	None
GSM8657563	DIvA cell, IDH mutant, non treated, TETdCD, over expression, 4C-seq, DSB1, Rep 1	Nov 27, 2025	approved	None
GSM8657564	DIvA cell, IDH mutant, non treated, TETdCD, over expression, 4C-seq, DSB1, Rep 2	Nov 27, 2025	approved	None
GSM8657565	DIvA cell, IDH mutant, non treated, empty vector, 4C-seq, DSB1, Rep 1	Nov 27, 2025	approved	None
GSM8657566	DIvA cell, IDH mutant, non treated, empty vector, 4C-seq, DSB1, Rep 2	Nov 27, 2025	approved	None
GSM8657567	DIvA cell, IDH mutant, non treated, Knock down (si1), 4C-seq, DSB1, Rep 1	Nov 27, 2025	approved	None
GSM8657568	DIvA cell, IDH mutant, non treated, Knock down (si1), 4C-seq, DSB1, Rep 2	Nov 27, 2025	approved	None
GSM8657569	DIvA cell, IDH mutant, non treated, Knock down (si2), 4C-seq, DSB1, Rep 1	Nov 27, 2025	approved	None
GSM8657570	DIvA cell, IDH mutant, non treated, Knock down (si2), 4C-seq, DSB1, Rep 2	Nov 27, 2025	approved	None
GSM8657571	DIvA cell, IDH wt, TETCD4-OHT, over expression, 4C-seq, DSB1, Rep 1	Nov 27, 2025	approved	None
GSM8657572	DIvA cell, IDH wt, TETCD4-OHT, over expression, 4C-seq, DSB1, Rep 2	Nov 27, 2025	approved	None
GSM8657573	DIvA cell, IDH wt, 4-OHT, Knock down (si1), 4C-seq, DSB1, Rep 1	Nov 27, 2025	approved	None
GSM8657574	DIvA cell, IDH wt, 4-OHT, Knock down (si1), 4C-seq, DSB1, Rep 2	Nov 27, 2025	approved	None
GSM8657575	DIvA cell, IDH wt, 4-OHT, Knock down (si2), 4C-seq, DSB1, Rep 1	Nov 27, 2025	approved	None
GSM8657576	DIvA cell, IDH wt, 4-OHT, Knock down (si2), 4C-seq, DSB1, Rep 2	Nov 27, 2025	approved	None
GSM8657577	DIvA cell, IDH wt, non treated, 4C-seq, DSB1, Rep 1	Nov 27, 2025	approved	None
GSM8657578	DIvA cell, IDH wt, non treated, 4C-seq, DSB1, Rep 2	Nov 27, 2025	approved	None
GSM8657579	DIvA cell, IDH wt, non treated, Knock down (si1), 4C-seq, DSB1, Rep 1	Nov 27, 2025	approved	None
GSM8657580	DIvA cell, IDH wt, non treated, Knock down (si1), 4C-seq, DSB1, Rep 2	Nov 27, 2025	approved	None
GSM8657581	DIvA cell, IDH wt, non treated, Knock down (si2), 4C-seq, DSB1, Rep 1	Nov 27, 2025	approved	None
GSM8657582	DIvA cell, IDH wt, non treated, Knock down (si2), 4C-seq, DSB1, Rep 2	Nov 27, 2025	approved	None
GSM8657583	DIvA cell, IDH mutant, TETCD4-OHT, over expression, 4C-seq, DSB2, Rep 1	Nov 27, 2025	approved	BW
GSM8657584	DIvA cell, IDH mutant, TETCD4-OHT, over expression, 4C-seq, DSB2, Rep 2	Nov 27, 2025	approved	BW
GSM8657585	DIvA cell, IDH mutant, 4-OHT, 4C-seq, DSB2, Rep 1	Nov 27, 2025	approved	BW
GSM8657586	DIvA cell, IDH mutant, 4-OHT, 4C-seq, DSB2, Rep 2	Nov 27, 2025	approved	BW
GSM8657587	DIvA cell, IDH mutant, TETdCD4-OHT, over expression, 4C-seq, DSB2, Rep 1	Nov 27, 2025	approved	BW
GSM8657588	DIvA cell, IDH mutant, TETdCD4-OHT, over expression, 4C-seq, DSB2, Rep 2	Nov 27, 2025	approved	BW
GSM8657589	DIvA cell, IDH mutant, 4-OHT, empty vector, 4C-seq, DSB2, Rep 1	Nov 27, 2025	approved	BW
GSM8657590	DIvA cell, IDH mutant, 4-OHT, empty vector, 4C-seq, DSB2, Rep 2	Nov 27, 2025	approved	BW
GSM8657591	DIvA cell, IDH mutant, 4-OHT, Knock down (si1), 4C-seq, DSB2, Rep 1	Nov 27, 2025	approved	BW
GSM8657592	DIvA cell, IDH mutant, 4-OHT, Knock down (si1), 4C-seq, DSB2, Rep 2	Nov 27, 2025	approved	BW
GSM8657593	DIvA cell, IDH mutant, 4-OHT, Knock down (si2), 4C-seq, DSB2, Rep 1	Nov 27, 2025	approved	BW
GSM8657594	DIvA cell, IDH mutant, 4-OHT, Knock down (si2), 4C-seq, DSB2, Rep 2	Nov 27, 2025	approved	BW
GSM8657595	DIvA cell, IDH mutant, non treated, 4-OHT, 4C-seq, DSB2, Rep 1	Nov 27, 2025	approved	None
GSM8657596	DIvA cell, IDH mutant, non treated, 4-OHT, 4C-seq, DSB2, Rep 2	Nov 27, 2025	approved	None
GSM8657597	DIvA cell, IDH mutatnt, non treated, TETCD, over expression, 4C-seq, DSB2, Rep 1	Nov 27, 2025	approved	None
GSM8657598	DIvA cell, IDH mutatnt, non treated, TETCD, over expression, 4C-seq, DSB2, Rep 2	Nov 27, 2025	approved	None
GSM8657599	DIvA cell, IDH mutatnt, non treated, TETdCD, over expression, 4C-seq, DSB2, Rep 1	Nov 27, 2025	approved	None
GSM8657600	DIvA cell, IDH mutant, non treated, TETdCD, over expression, 4C-seq, DSB2, Rep 2	Nov 27, 2025	approved	None
GSM8657601	DIvA cell, IDH mutant, non treated, empty vector, 4C-seq, DSB2, Rep 1	Nov 27, 2025	approved	None
GSM8657602	DIvA cell, IDH mutant, non treated, empty vector, 4C-seq, DSB2, Rep 2	Nov 27, 2025	approved	None
GSM8657603	DIvA cell, IDH mutant, non treated, Knock down (si1), 4C-seq, DSB2, Rep 1	Nov 27, 2025	approved	None
GSM8657604	DIvA cell, IDH mutant, non treated, Knock down (si1), 4C-seq, DSB2, Rep 2	Nov 27, 2025	approved	None
GSM8657605	DIvA cell, IDH mutant, non treated, Knock down (si2), 4C-seq, DSB2, Rep 1	Nov 27, 2025	approved	None
GSM8657606	DIvA cell, IDH mutant, non treated, Knock down (si2), 4C-seq, DSB2, Rep 2	Nov 27, 2025	approved	None
GSM8657607	DIvA cell, IDH wt, TETCD4-OHT, over expression, 4C-seq, DSB2, Rep 1	Nov 27, 2025	approved	None
GSM8657608	DIvA cell, IDH wt, TETCD4-OHT, over expression, 4C-seq, DSB2, Rep 2	Nov 27, 2025	approved	None
GSM8657609	DIvA cell, IDH wt, 4-OHT, Knock down (si1), 4C-seq, DSB2, Rep 1	Nov 27, 2025	approved	None
GSM8657610	DIvA cell, IDH wt, 4-OHT, Knock down (si1), 4C-seq, DSB2, Rep 2	Nov 27, 2025	approved	None
GSM8657611	DIvA cell, IDH wt, 4-OHT, Knock down (si2), 4C-seq, DSB2, Rep 1	Nov 27, 2025	approved	None
GSM8657612	DIvA cell, IDH wt, 4-OHT, Knock down (si2), 4C-seq, DSB2, Rep 2	Nov 27, 2025	approved	None
GSM8657613	DIvA cell, IDH wt, non treated, 4C-seq, DSB2, Rep 1	Nov 27, 2025	approved	None
GSM8657614	DIvA cell, IDH wt, non treated, 4C-seq, DSB2, Rep 2	Nov 27, 2025	approved	None
GSM8657615	DIvA cell, IDH wt, non treated, Knock down (si1), 4C-seq, DSB2, Rep 1	Nov 27, 2025	approved	None
GSM8657616	DIvA cell, IDH wt, non treated, Knock down (si1), 4C-seq, DSB2, Rep 2	Nov 27, 2025	approved	None
GSM8657617	DIvA cell, IDH wt, non treated, Knock down (si2), 4C-seq, DSB2, Rep 1	Nov 27, 2025	approved	None
GSM8657618	DIvA cell, IDH wt, non treated, Knock down (si2), 4C-seq, DSB2, Rep 2	Nov 27, 2025	approved	None

Research involving human participants, their data, or biological material

Policy information about studies with human participants or human data. See also policy information about sex, gender (identity/presentation), and sexual orientation and race, ethnicity and racism.

Reporting on sex and gender	N/A
Reporting on race, ethnicity, or other socially relevant groupings	N/A
Population characteristics	N/A
Recruitment	N/A
Ethics oversight	N/A

Note that full information on the approval of the study protocol must also be provided in the manuscript.

Field-specific reporting

Please select the one below that is the best fit for your research. If you are not sure, read the appropriate sections before making your selection.

[x] Life sciences [ ] Behavioural & social sciences [ ] Ecological, evolutionary & environmental sciences

For a reference copy of the document with all sections, see nature.com/documents/nr-reporting-summary-flat.pdf

Life sciences study design

All studies must disclose on these points even when the disclosure is negative.

Sample size	For statistical analysis, three biological replicates were conducted for each experiment group for in vitro assays. No statistical methods were used to determine sample size. For immunofluorescence quantification, at least 40 nuclei were analyzed for each condition.
Data exclusions	No data were excluded.
Replication	CTCF ChIP-seq in DIvA cells, two replicates. 4C-seq, two replicates. All the other experiments with quantifications were performed with three biological replicates.
Randomization	Cell cultures were randomly assigned to either experimental or control groups.
Blinding	N/A

Reporting for specific materials, systems and methods

We require information from authors about some types of materials, experimental systems and methods used in many studies. Here, indicate whether each material, system or method listed is relevant to your study. If you are not sure if a list item applies to your research, read the appropriate section before selecting a response.

Materials & experimental systems

n/a	Involved in the study	
[ ]	[x]	Antibodies
[ ]	[x]	Eukaryotic cell lines
[x]	[ ]	Palaeontology and archaeology
[x]	[ ]	Animals and other organisms
[x]	[ ]	Clinical data
[x]	[ ]	Dual use research of concern
[x]	[ ]	Plants

Methods

n/a	Involved in the study	
[ ]	[x]	ChIP-seq
[ ]	[x]	Flow cytometry
[x]	[ ]	MRI-based neuroimaging

Antibodies

Antibodies used	anti-5hmC antibody (Sigma, MABE176)<br>HRP-conjugated Goat anti-rabbit IgG secondary antibody (Thermo Fisher, 31410)<br>HRP-conjugated Goat anti-Mouse IgG (H+L) Secondary Antibody (Thermo Fisher, 31430)<br>Alexa Fluor 594 goat anti-mouse IgG (Thermo Fisher, A-11012)

	Alexa Fluor 488 goat anti-rabbit IgG antibodies (Thermo Fisher, A-11034)<br>RAD51 (Abcam, ab88572),<br>TET1 antibody [N3C1] (GeneTex, GTX124207)<br>TET2 Polyclonal Antibody (Thermo Fisher, PA5-78514)<br>anti-γH2A.X (Millipore, 05-636),<br>RAD51 (Cell Signaling Technology, ab88572),<br>BRCA2 (Bethyl Laboratories),<br>CTCF (Abcam, ab70303),<br>ATM S1981 (Cell Signaling Technology, 5883),<br>ATM (Cell Signaling Technology, 92356),<br>Anti-DDK (FLAG) monoclonal antibody (Origene, TA50011-1, Clone OTI4C5),<br>GAPDH (Cell Signaling Technology, 2118)<br><br>Antibody dilutions for western blots were as follows: Phospho-ATM (Ser1981) (D6H9) Rabbit mAb#5883 1:1000 in 5% BSA/PBST; ATM (Cell Signaling Technology, 92356, 1:1000 in 5% milk; Anti-DDK (FLAG) monoclonal antibody (Origene, TA50011-1, Clone OTI4C5), 1:1000 in 5% milk; TET1 antibody [N3C1] (GeneTex, GTX124207) and TET2 Polyclonal Antibody (Thermo Fisher, PA5-78514), 1/1000 in 5% milk; CTCF (Abcam, ab70303), RAD51 (Cell Signaling Technology, ab88572), 1/2000 in 5% milk; BRCA2 (Bethyl Laboratories), 1/500 in 5% milk; anti-γH2A.X (Millipore, 05-636), 1/1000 in 5% BSA/PBST; GAPDH (Cell Signaling Technology, 2118), 1:2000 in 5% milk; HRP-conjugated Goat anti-rabbit IgG secondary antibody (Thermo Fisher, 31410) and HRP-conjugated Goat anti-Mouse IgG (H +L) Secondary Antibody (Thermo Fisher, 31430) 1/6000 in 3% 5% BSA/PBST.<br>Antibody dilutions for Dot blot, anti-5hmC antibody (Sigma, MABE176, dilution 1:1,000)<br>Antibody dilutions for Immunofluorescence, CTCF (Abcam, ab70303), 1/1000 in 1% BSA/PBS; RAD51 (Cell Signaling Technology, ab88572), 1/1000 in 1% BSA/PBS; BRCA2 (Bethyl Laboratories), 1/500 in1% BSA/PBS; anti-γH2A.X (Millipore, 05-636), 1/1000 in 1% BSA/PBS; anti-5hmC antibody (Sigma, MABE176) 1/1000 in 1% BSA/PBS. Alexa Fluor 594 goat anti-mouse IgG (Thermo Fisher, A-11012) and Alexa Fluor 488 goat anti-rabbit IgG antibodies (Thermo Fisher, A-11034) 1/1000 in 1% BSA/PBS.<br>For ChIP-seq, 2 ug of CTCF (Abcam, ab70303) antibodies were diluted in 200 ul reaction solutions.<br>Antibody dilution in Proximity Ligation Assay (PLA), CTCF (Abcam, ab70303), RAD51 (Abcam, ab88572), and BRCA2 (Bethyl Laboratories, A303-434A) were used at 1:1,000 dilution.
Validation	For those commercial antibodies, the validation statements are provided on the manufacturer's websites.<br>TET1 and TET2 antibody were validated by knockout data provided in Figure 1 in this manuscript.<br>CTCF antibodies were validated by knock down data provided in Extended data Figure 3 in this manuscript; IP data in Figure 4 and Extended data Figure 4.<br>RAD51 and BRCA2 antibody were validated by the IP data in Figure 4 and Extended data Figure 4.

Eukaryotic cell lines

Policy information about cell lines and Sex and Gender in Research

Cell line source(s)	The U251 cell line was obtained from Sigma Aldrich.<br>The U2OS DRGFP cell line was purchased from the ATCC. To generate the inducible Sce-I DRGFP cell line, the fragment of Shield1 ligand-dependent destabilization domain (DD), Sce-I and glucocorticoid receptor (GR) was cloned to the pLenti-C-Myc-DDK-IRES-BSD Lentiviral Gene Expression Vector with Mlu I and Xho I restriction enzyme sites. U2OS DRGFP cells were infected with the lentivirus expressing DD-SceI-GR protein.<br>DIvA cells were obtained from Dr. Legube's laboratory. IDH-wild type or IDH R132H mutant DIvA cells were generated with lentivirus infection.
Authentication	Authentication of the U251 and U2OS cell lines were performed by the provider ATCC and European Collection of Authenticated Cell Cultures (ECACC) through morphology, karyotyping and short tandem repeat (STR)-PCR profiling-based approaches to confirm the identity of these cells.
Mycoplasma contamination	Cells were routinely tested for mycoplasma and were tested negative.
Commonly misidentified lines<br>(See ICLAC register)	No

Plants

Seed stocks	N/A
Novel plant genotypes	N/A
Authentication	N/A

ChIP-seq

Data deposition

☒ Confirm that both raw and final processed data have been deposited in a public database such as GEO.

☒ Confirm that you have deposited or provided access to graph files (e.g. BED files) for the called peaks.

Data access links
May remain private before publication.

Our sequencing data from ChIP-seq and 4C-seq have been deposited in the Gene Expression Omnibus and the accession number is GSE283196 and the reviewer token is: mvglmkimrrczviz

Files in database submission

GSE283196 CTCF distribution around AsisI sites in IDHwt and IDHR132H DIvA cells Nov 27, 2025 approved None
GSM8657501 DIvA cell, IDH wt, non treated, CTCF, ChIP, Rep 1 Nov 27, 2025 approved BW NARROWPEAK
GSM8657502 DIvA cell, IDH wt, non treated, CTCF, ChIP, Rep 2 Nov 27, 2025 approved BW NARROWPEAK
GSM8657503 DIvA cell, IDH wt, non treated, CTCF, ChIP, Rep 3 Nov 27, 2025 approved BW NARROWPEAK
GSM8657504 DIvA cell, IDH wt, non treated, CTCF, ChIP, Rep 4 Nov 27, 2025 approved BW NARROWPEAK
GSM8657506 DIvA cell, IDH wt, 4-OHT, CTCF, Rep 1 Nov 27, 2025 approved BW NARROWPEAK
GSM8657507 DIvA cell, IDH wt, 4-OHT, CTCF, Rep 2 Nov 27, 2025 approved BW NARROWPEAK
GSM8657508 DIvA cell, IDH wt, 4-OHT, CTCF, Rep 3 Nov 27, 2025 approved BW NARROWPEAK
GSM8657509 DIvA cell, IDH wt, 4-OHT, CTCF, Rep 4 Nov 27, 2025 approved BW NARROWPEAK
GSM8657510 DIvA cell, IDH mutant, non treated, CTCF, Rep 1 Nov 27, 2025 approved BW NARROWPEAK
GSM8657511 DIvA cell, IDH mutant, non treated, CTCF, Rep 2 Nov 27, 2025 approved BW NARROWPEAK
GSM8657512 DIvA cell, IDH mutant, non treated, CTCF, Rep 3 Nov 27, 2025 approved BW NARROWPEAK
GSM8657513 DIvA cell, IDH mutant, non treated, CTCF, Rep 4 Nov 27, 2025 approved BW NARROWPEAK
GSM8657514 DIvA cell, IDH mutant, 4-OHT,CTCF, Rep 1 Nov 27, 2025 approved BW NARROWPEAK
GSM8657515 DIvA cell, IDH mutant, 4-OHT,CTCF, Rep 2 Nov 27, 2025 approved BW NARROWPEAK
GSM8657516 DIvA cell, IDH mutant, 4-OHT,CTCF, Rep 3 Nov 27, 2025 approved BW NARROWPEAK
GSM8657517 DIvA cell, IDH mutant, 4-OHT,CTCF, Rep 4 Nov 27, 2025 approved BW NARROWPEAK
GSM8657518 DIvA cell, IDH mutant, non treated, TETCD, Rep1 Nov 27, 2025 approved BW NARROWPEAK
GSM8657519 DIvA cell, IDH mutant, non treated, TETCD, Rep2 Nov 27, 2025 approved BW NARROWPEAK
GSM8657520 DIvA cell, IDH mutant, TETCD4-OHT over expression, Rep1 Nov 27, 2025 approved BW NARROWPEAK
GSM8657521 DIvA cell, IDH mutant, TETCD4-OHT over expression, Rep2 Nov 27, 2025 approved BW NARROWPEAK
GSM8657522 DIvA cell, IDH mutant, non treated, TETdCD, Rep1 Nov 27, 2025 approved BW NARROWPEAK
GSM8657523 DIvA cell, IDH mutant, non treated, TETdCD, Rep2 Nov 27, 2025 approved BW NARROWPEAK
GSM8657524 DIvA cell, IDH mutant, non treated, TETdCD, Rep3 Nov 27, 2025 approved BW NARROWPEAK
GSM8657525 DIvA cell, IDH mutant, TETdCD4-OHT over expression, Rep1 Nov 27, 2025 approved BW NARROWPEAK
GSM8657526 DIvA cell, IDH mutant, TETdCD4-OHT over expression, Rep2 Nov 27, 2025 approved BW NARROWPEAK
GSM8657527 DIvA cell, IDH wt, non treated, input, Rep 1 Nov 27, 2025 approved None
GSM8657528 DIvA cell, IDH wt, non treated, input, Rep 2 Nov 27, 2025 approved None
GSM8657529 DIvA cell, IDH wt, non treated, input, Rep 3 Nov 27, 2025 approved None
GSM8657530 DIvA cell, IDH wt, non treated, input, Rep 4 Nov 27, 2025 approved None
GSM8657531 DIvA cell, IDH wt, 4-OHT, input, Rep 1 Nov 27, 2025 approved None
GSM8657532 DIvA cell, IDH wt, 4-OHT, input, Rep 2 Nov 27, 2025 approved None
GSM8657533 DIvA cell, IDH wt, 4-OHT, input, Rep 3 Nov 27, 2025 approved None
GSM8657534 DIvA cell, IDH wt, 4-OHT, input, Rep 4 Nov 27, 2025 approved None
GSM8657535 DIvA cell, IDH mutant, non treated, input, Rep 1 Nov 27, 2025 approved None
GSM8657536 DIvA cell, IDH mutant, non treated, input, Rep 2 Nov 27, 2025 approved None
GSM8657537 DIvA cell, IDH mutant, 4-OHT, input, Rep 1 Nov 27, 2025 approved None
GSM8657538 DIvA cell, IDH mutant, 4-OHT, input, Rep 2 Nov 27, 2025 approved None
GSM8657539 DIvA cell, IDH mutant, non treated, TETCD, input, Rep1 Nov 27, 2025 approved None
GSM8657540 DIvA cell, IDH mutant, non treated, TETCD, input, Rep2 Nov 27, 2025 approved None
GSM8657541 DIvA cell, IDH mutant, TETCD4-OHT over expression, input, Rep1 Nov 27, 2025 approved None
GSM8657542 DIvA cell, IDH mutant, TETCD4-OHT over expression, input, Rep2 Nov 27, 2025 approved None
GSM8657543 DIvA cell, IDH mutant, non treated, TETdCD, input, Rep1 Nov 27, 2025 approved None
GSM8657544 DIvA cell, IDH mutant, non treated, TETdCD, input, Rep2 Nov 27, 2025 approved None
GSM8657545 DIvA cell, IDH mutant, TETdCD4-OHT over expression, input, Rep1 Nov 27, 2025 approved None
GSM8657546 DIvA cell, IDH mutant, TETdCD4-OHT over expression, input, Rep2 Nov 27, 2025 approved None
GSM8657547 DIvA cell, IDH mutant, TETCD4-OHT, over expression, 4C-seq, DSB1, Rep 1 Nov 27, 2025 approved BW
GSM8657548 DIvA cell, IDH mutant, TETCD4-OHT, over expression, 4C-seq, DSB1, Rep 2 Nov 27, 2025 approved BW
GSM8657549 DIvA cell, IDH mutant, 4-OHT, 4C-seq, DSB1, Rep 1 Nov 27, 2025 approved BW
GSM8657550 DIvA cell, IDH mutant, 4-OHT, 4C-seq, DSB1, Rep 2 Nov 27, 2025 approved BW
GSM8657551 DIvA cell, IDH mutant, TETdCD4-OHT, over expression, 4C-seq, DSB1, Rep 1 Nov 27, 2025 approved BW
GSM8657552 DIvA cell, IDH mutant, TETdCD4-OHT, over expression, 4C-seq, DSB1, Rep 2 Nov 27, 2025 approved BW
GSM8657553 DIvA cell, IDH mutant, TETdCD4-OHT, empty vector, 4C-seq, DSB1, Rep 1 Nov 27, 2025 approved BW
GSM8657554 DIvA cell, IDH mutant, TETdCD4-OHT, empty vector, 4C-seq, DSB1, Rep 2 Nov 27, 2025 approved BW
GSM8657555 DIvA cell, IDH mutant, TETdCD4-OHT, Knock down (si1), 4C-seq, DSB1, Rep 1 Nov 27, 2025 approved BW
GSM8657556 DIvA cell, IDH mutant, TETdCD4-OHT, Knock down (si1), 4C-seq, DSB1, Rep 2 Nov 27, 2025 approved BW
GSM8657557 DIvA cell, IDH mutant, TETdCD4-OHT, Knock down (si2), 4C-seq, DSB1, Rep 1 Nov 27, 2025 approved BW

GSM8657558 DIvA cell, IDH mutant, TETdCD4-OHT, Knock down (si2), 4C-seq, DSB1, Rep 2 Nov 27, 2025 approved BW
GSM8657559 DIvA cell, IDH mutant, non treated, 4C-seq, DSB1, Rep 1 Nov 27, 2025 approved None
GSM8657560 DIvA cell, IDH mutant, non treated, 4C-seq, DSB1, Rep 2 Nov 27, 2025 approved None
GSM8657561 DIvA cell, IDH mutant, non treated, TETCD, over expression, 4C-seq, DSB1, Rep 1 Nov 27, 2025 approved None
GSM8657562 DIvA cell, IDH mutant, non treated, TETCD, over expression, 4C-seq, DSB1, Rep 2 Nov 27, 2025 approved None
GSM8657563 DIvA cell, IDH mutant, non treated, TETdCD, over expression, 4C-seq, DSB1, Rep 1 Nov 27, 2025 approved None
GSM8657564 DIvA cell, IDH mutant, non treated, TETdCD, over expression, 4C-seq, DSB1, Rep 2 Nov 27, 2025 approved None
GSM8657565 DIvA cell, IDH mutant, non treated, empty vector, 4C-seq, DSB1, Rep 1 Nov 27, 2025 approved None
GSM8657566 DIvA cell, IDH mutant, non treated, empty vector, 4C-seq, DSB1, Rep 2 Nov 27, 2025 approved None
GSM8657567 DIvA cell, IDH mutant, non treated, Knock down (si1), 4C-seq, DSB1, Rep 1 Nov 27, 2025 approved None
GSM8657568 DIvA cell, IDH mutant, non treated, Knock down (si1), 4C-seq, DSB1, Rep 2 Nov 27, 2025 approved None
GSM8657569 DIvA cell, IDH mutant, non treated, Knock down (si2), 4C-seq, DSB1, Rep 1 Nov 27, 2025 approved None
GSM8657570 DIvA cell, IDH mutant, non treated, Knock down (si2), 4C-seq, DSB1, Rep 2 Nov 27, 2025 approved None
GSM8657571 DIvA cell, IDH wt, TETCD4-OHT, over expression, 4C-seq, DSB1, Rep 1 Nov 27, 2025 approved None
GSM8657572 DIvA cell, IDH wt, TETCD4-OHT, over expression, 4C-seq, DSB1, Rep 2 Nov 27, 2025 approved None
GSM8657573 DIvA cell, IDH wt, 4-OHT, Knock down (si1), 4C-seq, DSB1, Rep 1 Nov 27, 2025 approved None
GSM8657574 DIvA cell, IDH wt, 4-OHT, Knock down (si1), 4C-seq, DSB1, Rep 2 Nov 27, 2025 approved None
GSM8657575 DIvA cell, IDH wt, 4-OHT, Knock down (si2), 4C-seq, DSB1, Rep 1 Nov 27, 2025 approved None
GSM8657576 DIvA cell, IDH wt, 4-OHT, Knock down (si2), 4C-seq, DSB1, Rep 2 Nov 27, 2025 approved None
GSM8657577 DIvA cell, IDH wt, non treated, 4C-seq, DSB1, Rep 1 Nov 27, 2025 approved None
GSM8657578 DIvA cell, IDH wt, non treated, 4C-seq, DSB1, Rep 2 Nov 27, 2025 approved None
GSM8657579 DIvA cell, IDH wt, non treated, Knock down (si1), 4C-seq, DSB1, Rep 1 Nov 27, 2025 approved None
GSM8657580 DIvA cell, IDH wt, non treated, Knock down (si1), 4C-seq, DSB1, Rep 2 Nov 27, 2025 approved None
GSM8657581 DIvA cell, IDH wt, non treated, Knock down (si2), 4C-seq, DSB1, Rep 1 Nov 27, 2025 approved None
GSM8657582 DIvA cell, IDH wt, non treated, Knock down (si2), 4C-seq, DSB1, Rep 2 Nov 27, 2025 approved None
GSM8657583 DIvA cell, IDH mutant, TETCD4-OHT, over expression, 4C-seq, DSB2, Rep 1 Nov 27, 2025 approved BW
GSM8657584 DIvA cell, IDH mutant, TETCD4-OHT, over expression, 4C-seq, DSB2, Rep 2 Nov 27, 2025 approved BW
GSM8657585 DIvA cell, IDH mutant, 4-OHT, 4C-seq, DSB2, Rep 1 Nov 27, 2025 approved BW
GSM8657586 DIvA cell, IDH mutant, 4-OHT, 4C-seq, DSB2, Rep 2 Nov 27, 2025 approved BW
GSM8657587 DIvA cell, IDH mutant, TETdCD4-OHT, over expression, 4C-seq, DSB2, Rep 1 Nov 27, 2025 approved BW
GSM8657588 DIvA cell, IDH mutant, TETdCD4-OHT, over expression, 4C-seq, DSB2, Rep 2 Nov 27, 2025 approved BW
GSM8657589 DIvA cell, IDH mutant, 4-OHT, empty vector, 4C-seq, DSB2, Rep 1 Nov 27, 2025 approved BW
GSM8657590 DIvA cell, IDH mutant, 4-OHT, empty vector, 4C-seq, DSB2, Rep 2 Nov 27, 2025 approved BW
GSM8657591 DIvA cell, IDH mutant, 4-OHT, Knock down (si1), 4C-seq, DSB2, Rep 1 Nov 27, 2025 approved BW
GSM8657592 DIvA cell, IDH mutant, 4-OHT, Knock down (si1), 4C-seq, DSB2, Rep 2 Nov 27, 2025 approved BW
GSM8657593 DIvA cell, IDH mutant, 4-OHT, Knock down (si2), 4C-seq, DSB2, Rep 1 Nov 27, 2025 approved BW
GSM8657594 DIvA cell, IDH mutant, 4-OHT, Knock down (si2), 4C-seq, DSB2, Rep 2 Nov 27, 2025 approved BW
GSM8657595 DIvA cell, IDH mutant, non treated, 4-OHT, 4C-seq, DSB2, Rep 1 Nov 27, 2025 approved None
GSM8657596 DIvA cell, IDH mutant, non treated, 4-OHT, 4C-seq, DSB2, Rep 2 Nov 27, 2025 approved None
GSM8657597 DIvA cell, IDH mutatnt, non treated, TETCD, over expression, 4C-seq, DSB2, Rep 1 Nov 27, 2025 approved None
GSM8657598 DIvA cell, IDH mutatnt, non treated, TETCD, over expression, 4C-seq, DSB2, Rep 2 Nov 27, 2025 approved None
GSM8657599 DIvA cell, IDH mutatnt, non treated, TETdCD, over expression, 4C-seq, DSB2, Rep 1 Nov 27, 2025 approved None
GSM8657600 DIvA cell, IDH mutant, non treated, TETdCD, over expression, 4C-seq, DSB2, Rep 2 Nov 27, 2025 approved None
GSM8657601 DIvA cell, IDH mutant, non treated, empty vector, 4C-seq, DSB2, Rep 1 Nov 27, 2025 approved None
GSM8657602 DIvA cell, IDH mutant, non treated, empty vector, 4C-seq, DSB2, Rep 2 Nov 27, 2025 approved None
GSM8657603 DIvA cell, IDH mutant, non treated, Knock down (si1), 4C-seq, DSB2, Rep 1 Nov 27, 2025 approved None
GSM8657604 DIvA cell, IDH mutant, non treated, Knock down (si1), 4C-seq, DSB2, Rep 2 Nov 27, 2025 approved None
GSM8657605 DIvA cell, IDH mutant, non treated, Knock down (si2), 4C-seq, DSB2, Rep 1 Nov 27, 2025 approved None
GSM8657606 DIvA cell, IDH mutant, non treated, Knock down (si2), 4C-seq, DSB2, Rep 2 Nov 27, 2025 approved None
GSM8657607 DIvA cell, IDH wt, TETCD4-OHT, over expression, 4C-seq, DSB2, Rep 1 Nov 27, 2025 approved None
GSM8657608 DIvA cell, IDH wt, TETCD4-OHT, over expression, 4C-seq, DSB2, Rep 2 Nov 27, 2025 approved None
GSM8657609 DIvA cell, IDH wt, 4-OHT, Knock down (si1), 4C-seq, DSB2, Rep 1 Nov 27, 2025 approved None
GSM8657610 DIvA cell, IDH wt, 4-OHT, Knock down (si1), 4C-seq, DSB2, Rep 2 Nov 27, 2025 approved None
GSM8657611 DIvA cell, IDH wt, 4-OHT, Knock down (si2), 4C-seq, DSB2, Rep 1 Nov 27, 2025 approved None
GSM8657612 DIvA cell, IDH wt, 4-OHT, Knock down (si2), 4C-seq, DSB2, Rep 2 Nov 27, 2025 approved None
GSM8657613 DIvA cell, IDH wt, non treated, 4C-seq, DSB2, Rep 1 Nov 27, 2025 approved None
GSM8657614 DIvA cell, IDH wt, non treated, 4C-seq, DSB2, Rep 2 Nov 27, 2025 approved None
GSM8657615 DIvA cell, IDH wt, non treated, Knock down (si1), 4C-seq, DSB2, Rep 1 Nov 27, 2025 approved None
GSM8657616 DIvA cell, IDH wt, non treated, Knock down (si1), 4C-seq, DSB2, Rep 2 Nov 27, 2025 approved None
GSM8657617 DIvA cell, IDH wt, non treated, Knock down (si2), 4C-seq, DSB2, Rep 1 Nov 27, 2025 approved None
GSM8657618 DIvA cell, IDH wt, non treated, Knock down (si2), 4C-seq, DSB2, Rep 2 Nov 27, 2025 approved None

Genome browser session (e.g. UCSC)	UCSC

Methodology

Replicates	CTCF ChIP-seq, before and after DSB induction, 2-4 replicates<br>4C-seq before and after DSB induction, 2 replicates
Sequencing depth	ChIP-seq, 30M reads per condition<br>4C-seq, 20M reads per condition
Antibodies	CTCF (Abcam, ab70303),
Peak calling parameters	CTCF peaks were identified using MACS2 program with callpeak algorithm, with default setting. Input was used as the control.<br>macs2 callpeak -t <input file.bam> -c <control file.bam> -n <output file> [--nolambda]
Data quality	All sequencing data underwent FastQC analysis. During the SAM to BAM conversion step, sequencing reads were filtered based on their Phred score (-q 20 or 25).
Software	Bowtie2-2.5.1<br>MACS2-2.7.1<br>4Cpipe tool<br>Peak C R package, version R/4.2<br>GraphPad Prism 8.4.3

Flow Cytometry

Plots

Confirm that:

- [x] The axis labels state the marker and fluorochrome used (e.g. CD4-FITC).
- [x] The axis scales are clearly visible. Include numbers along axes only for bottom left plot of group (a 'group' is an analysis of identical markers).
- [x] All plots are contour plots with outliers or pseudocolor plots.
- [x] A numerical value for number of cells or percentage (with statistics) is provided.

Methodology

Sample preparation	The DD-sce-GR / NHEJ reporter Cells were collected and washed with PBS. Then cells were filtered through a nylon mesh cell strainer snap cap before detection with BD Accuri C6 flow cytometer or FACSCanto II.
Instrument	BD Accuri C6 flow cytometer and FACSCanto II.
Software	Data was acquired with BD FACSDiva software and analyzed with Flowjo v10.7.2.
Cell population abundance	DD-sce-GR / NHEJ reporter cells were divided into GFP-positive and GFP-negative populations subsequent to treatment with 0.5 µM Shield1 and 0.2 mM Triamcinolone acetonide (TA). The GFP-positive cell subset was characterized by GFP expression exceeding an intensity of 2x10^3 and quantified using FlowJo.
Gating strategy	The GFP-positive cell subset was characterized by GFP expression exceeding an intensity of 2x10^3. This gating strategy is shown in the supplementary Information. A negative control sample confirmed this gating strategy.

- [x] Tick this box to confirm that a figure exemplifying the gating strategy is provided in the Supplementary Information.
